# NMAstudio 2.0: An interactive tool for network meta-analysis to enhance understanding, interpretation, and communication of the findings

**DOI:** 10.1017/rsm.2026.10074

**Published:** 2026-03-06

**Authors:** Tianqi Yu, Silvia Metelli, Theodoros Papakonstantinou, Anna Chaimani

**Affiliations:** 1 Center of Research in Epidemiology and Statistics (CRESS-U1153), Université Paris Cité, Inserm, France; 2 Laboratory of Hygiene, Social & Preventive Medicine and Medical Statistics, Aristotle University of Thessaloniki School of Medicine, Greece; 3 Oslo Center for Biostatistics and Epidemiology, Department of Biostatistics, University of Oslo, Norway

**Keywords:** comparative effectiveness research, data synthesis, interactive visualizations, multiple treatments, open-access software

## Abstract

Network meta-analysis (NMA) is a vital methodology for synthesizing evidence across multiple treatments and informing medical decision-making. However, effective visualization and interpretation of results from large networks of interventions remain challenging, particularly for non-specialists. NMAstudio 2.0 is an innovative, interactive web application designed to address these difficulties by streamlining NMA workflows and enhancing result visualization. Developed using Python and R, NMAstudio 2.0 seamlessly integrates with established NMA frameworks. Our exemplar application of NMAstudio 2.0 using a Cochrane Review comparing several treatments for chronic plaque psoriasis demonstrates its capacity to facilitate all crucial steps of an NMA. The application features an intuitive interface for uploading data, automating analyses, generating interactive visualizations such as network diagrams, forest plots, ranking plots, and producing unique outputs like boxplots for transitivity checks and bidimensional forest plots. Most outputs are dynamically linked with the network diagram, enabling users to interactively explore evidence networks, apply advanced filtering, and highlight specific features by selecting nodes or edges within the diagram. While NMAstudio 2.0 aims to simplify NMAs, it also incorporates steps during the data upload process to mitigate the risk of producing poorly reported NMAs. NMAstudio 2.0 represents a significant step forward in improving the usability and accessibility of NMA, offering researchers a robust, versatile platform for evidence synthesis. Its integration of advanced features with an emphasis on user experience positions it as a valuable resource for enhancing decision-making and promoting evidence-based practice across diverse contexts.

## Highlights

### What is already known?

- Network meta-analysis (NMA) is a crucial method for synthesizing evidence across multiple treatments and guiding medical decision-making.
- Visualizing and interpreting results from large networks of interventions remain challenging, especially for non-specialists.

### What is new?

- NMAstudio 2.0 is an interactive web application designed to simplify NMA workflows and improve result visualization.
- It features a user-friendly interface for uploading data, automating analyses, and creating interactive visualizations.
- Outputs are dynamically linked to the network diagram, enabling users to explore evidence networks, apply filters, and highlight key features interactively.
- To support open research, NMAstudio 2.0 includes functions for saving and sharing uploaded data, making it easier for users to manage and share research projects.

### Potential impact for research synthesis methods readers

- NMAstudio 2.0 enhances understanding and interpretation of results, accounting for risks of bias and data limitations, while fostering data sharing and open science.
- Its advanced features and focus on usability make it a valuable tool for evidence-based decision-making across various research contexts.

## 1 Background

Network meta-analysis (NMA) is a powerful tool for synthesizing evidence on multiple healthcare interventions and is widely used to support clinical recommendations and guidelines. By combining for every comparison evidence that comes directly and indirectly from the available studies within a connected network of interventions, it usually offers more comprehensive and precise findings than individual studies or pairwise meta-analysis do.^1^ Beyond estimating relative effects, NMA offers the ability to rank the competing interventions based on specific outcomes and to facilitate decision-making, particularly in the presence of many alternative interventions for the condition of interest.

Despite the growing popularity of NMAs and the availability of extensive guidance, criticisms persist mainly due to their complex methodology and assumptions, which are not always examined carefully.^2^^–^ ^5^ Several empirical studies have identified deficiencies in published NMAs, although an improvement in the overall quality has been observed over time.^6^^,^ ^7^ The significant level of statistical expertise required and the long outputs that NMA typically produces pose a major barrier in understanding and properly considering the strengths and limitations of the findings.^2^ Additionally, even though several software tools are available for conducting NMAs,^8^^,^ ^9^ they do not offer visualizations that allow to easily make the connection between data and results.

Complete reporting is a crucial aspect of every NMA to avoid misinterpretations or over-confidence in uncertain findings. The *PRISMA-NMA* checklist^10^ assists researchers toward this direction, but often it is only partially followed by published NMAs. On the other hand, comprehensive NMAs usually put a lot of the required information into Supplementary Materials, which can extend to hundreds of pages.^11^^,^ ^12^ Navigating through these extensive and complex outputs can be both time-consuming and confusing, thereby impeding effective communication and decision-making. Typically, the volume of information in an NMA is significantly greater than that of a pairwise meta-analysis. This presents significant challenges to the principles of open science, which emphasize transparency, accessibility, and reproducibility.^13^^,^ ^14^ since it makes it difficult to share data and findings in a user-friendly manner.

In recent years, several new graphical and tabular visualizations for NMA have been introduced.^15^^,^ ^16^ For example, contribution plots highlight the balance between direct and indirect information in the results;^17^ radial ranking plots integrate network graphs with the relative ranking of the interventions;^18^ and the Kilim plot depicts absolute risk for each outcome and treatment alongside the strength of statistical evidence.^19^ However, all these visualizations typically need to be run separately in a scripting environment such as R or STATA and thus lack interactivity and interoperability.

In this paper, we introduce NMAstudio 2.0, an online interactive user interface for conducting, reviewing, and managing network meta-analyses. Compared to existing applications, NMAstudio 2.0 integrates several innovative features such as transitivity assessment via box plots and scatter plots, analysis and visualizations for multiple outcomes, and interactive graphics that make the complexity of NMAs more accessible and transparent. Through novel visualizations, our tool enhances understanding and interpretation of findings in light of risk of bias and other potential limitations of the data, while promoting data sharing and open science. At the same time, it supports transparency by guiding researchers to go through all the required steps for a complete report. NMAstudio 2.0 also simplifies the full NMA process by offering a “point-and-click” environment that completely removes the need for scripting. In the rest of the article, we first describe the main characteristics of the tool, and we introduce our illustrative example dataset. Then, we go through the different steps of a typical NMA and show how NMAstudio 2.0 can address existing challenges in the entire process. A brief description of the key terms used throughout the paper is available in **Box 1**.

Box 1.
Explanation of key NMA terms.

**PRISMA-NMA**

PRISMA-NMA is an extension of the PRISMA statement, created specifically to improve the reporting of systematic reviews that incorporate network meta-analyses (NMA). This extension includes a 32-item checklist, developed to address key elements considered essential for the accurate and thorough reporting of NMAs.

**Transitivity**

Transitivity is a key assumption in network meta-analysis that ensures the validity of indirect comparisons. It requires that studies comparing different interventions are similar, on average, with respect to all potential effect modifiers. It also implies that intervention A, as assessed in A versus B studies, is equivalent to intervention A in A versus C studies.

**Prediction interval**

A prediction interval is the interval within which the underlying effect of a new study similar to those included in a meta-analysis is expected to lie. Prediction intervals provide a way to assess the impact of the estimated heterogeneity and its uncertainty on the findings of the meta-analysis.

**CINeMA**

CINeMA (confidence in network meta-analysis) is a methodological framework for evaluating the confidence (or certainty) of the results from network meta-analyses. A key element of CINeMA is the percentage contribution matrix, which indicates the proportion of information contributed by each study or direct comparison to each network estimate.

**P-score**

The P-score is a statistical measure used to rank treatments based on a specific outcome and shows the mean extent of certainty that a treatment is better than its competitors. It is calculated using the probabilities that one treatment is better than another, considering all direct and indirect comparisons within the network. These are determined based on the estimated relative effects and their variance.

**Consistency**

The consistency assumption refers to the agreement between different sources of evidence—direct and indirect—in a network meta-analysis. It reflects the assumption of transitivity, which considers potential clinical and methodological variations across comparisons. Consistency is assessed through statistical tests, such as the design-by-treatment interaction model and the side-splitting approach. The former assesses consistency in the entire network, whereas the latter at the comparison level.

**Comparison-adjusted funnel plot**

A comparison-adjusted funnel plot is a variation of the standard funnel plot used in network meta-analysis to assess small-study effects while accounting for multiple comparisons. The horizontal axis shows the difference between study-specific effects and the corresponding comparison-specific summary effect, while the vertical axis represents the precision of the study, often measured by the inverse of its standard error. For proper interpretation, comparisons should be defined consistently (e.g., placebo versus active treatment). A symmetric comparison-adjusted funnel plot around the zero line indicates no indication of small-study effects.

## 2 Implementation

NMAstudio 2.0 is a Python web application integrated into the Python “Dash”^20^ environment and further connected to the R packages “netmeta”^21^ for NMA and “meta”^22^ for pairwise meta-analysis. NMA results are imported from R into Python, where interactive and downloadable visualizations are created using “Plotly” modules. Users of NMAstudio 2.0 do not need any prior familiarity with these software packages. The application is open-source and accessible via a web browser at https://nmastudio.uiocloud.no/. The source code and data are available in the following GitHub repository (https://github.com/CER-METHODS/NMAstudio-2.0) under the MIT license. We also include instructions on how to self-host the application for users with data privacy concerns, although the process is not trivial.

Currently, NMAstudio 2.0 consists of three pages (see screenshots in the Supplementary file): the homepage, the analysis setup page, and the results page. In the analysis setup page, users upload their data and specify the variables that represent outcomes, risk of bias, and potential effect modifiers. Once these steps are completed, the analysis is performed using the R netmeta package,^21^ and additional customizable visualizations are generated automatically. The results page then displays all produced plots and tables in an interactive format. Users can explore data and results by interacting with all the outputs, or can export them and prepare their NMA report.

## 3 Results

### 3.1 Illustrative example

To illustrate the use and the benefits of our tool, we use throughout this manuscript an exemplar NMA comparing systemic pharmacological treatments for chronic plaque psoriasis.^23^ The dataset contains 158 randomized controlled trials (RCTs) involving 20 treatments, including placebo. The full names and abbreviations of the treatments are provided in the Supplementary file. This NMA focuses on eight outcomes (two primary and six secondary). For our illustration, we will use four outcomes:

Primary outcomes:

- Efficacy: Proportion of participants achieving psoriasis area severity index ≥90 (PASI 90)
- Safety: The proportions of participants with serious adverse events (SAE)

Secondary outcomes:

- Quality of life measured by the dermatology life quality index (DLQI).
- The proportions of participants with any kind of adverse events (AE).

PASI90, SAE, and AE are binary outcomes, and DLQI is a continuous outcome. Example data for these four outcomes are provided in the Supplementary file.

For all binary outcomes, we use the risk ratio (RR) as the effect measure; for the continuous outcome, the standardized mean difference (SMD) is used due to the presence of different scales. The main interest here lies in the comparisons between the different active drugs rather than comparisons against placebo. However, placebo-controlled trials provide a large amount of data (58% of the trials) and thus have been included in the network to supplement the analysis.

### 3.2 Uploading the data

NMAstudio 2.0 streamlines the NMA process with an interactive interface that lets users upload datasets and run analyses through simple point-and-click actions, guided by embedded instructions. It supports three data formats—long, wide, or contrast data (see the Supplementary file for an explanation of the different data formats)—and allows users to upload data for several outcomes. Users need to follow the instructions on the data upload page and select a few options. Then, NMAstudio 2.0 automatically runs the analysis, generates results, and displays key graphs and tables. Step-by-step guidance for the general data upload process is available on the *Setup Analysis* page by clicking the “See here explanations for each step” button, and instructions for the illustrative data are provided in the Supplementary file (Illustrative_data_upload_instruction.pdf).

While NMAstudio 2.0 provides flexibility in data uploading, it also involves steps that reassure a comprehensive and transparent reporting. Specifically, users are recommended to include study risk of bias information and provide variables representing potential effect modifiers in their data, which are essential for evaluating the credibility of NMA results. A further crucial aspect of high-quality systematic reviews is the development of a protocol that outlines the main objectives, study design, and planned analyses of the review.^24^ Registering the protocol is important for ensuring transparency, reducing reporting bias, avoiding data-driven decisions, and preventing the duplication of reviews.^25^ Therefore, NMAstudio 2.0 asks users to provide the link to their protocol, which will then appear on the results page. This allows all end-users as well as peer-reviewers to easily access and compare the final review with the original protocol.^26^ If information on study risk of bias, potential effect modifiers, or the protocol link is not provided, warnings appear on the results page (Supplementary Figure S1). All settings for the statistical analysis can be found on the results page by clicking the “Download settings of statistical analysis” button. Additional warnings related to the dataset may appear during the data checks, but these do not appear on the results page (see Supplementary Figure S2 and the associated explanations in the Supplementary File).

### 3.3 Exploring the structure of the data

A powerful feature of the application is the interactive network diagram. The function of the graph is to give an outline of the structure of the data and the included studies. It also acts as a global filter and selector for the rest of the application. The graph is linked with an adjacent table of the selected studies. In the graph, each node represents an intervention, while each edge represents a direct comparison. Users can select interventions or comparisons by clicking on the corresponding nodes or edges. When a node is selected, the table highlights only the treatments that are directly compared with the selected intervention, helping users quickly identify comparisons supported by direct evidence from the available studies. Once nodes or edges are selected, the rest of the visualizations will display information specific to the corresponding interventions and comparisons.

**Figure 1:**
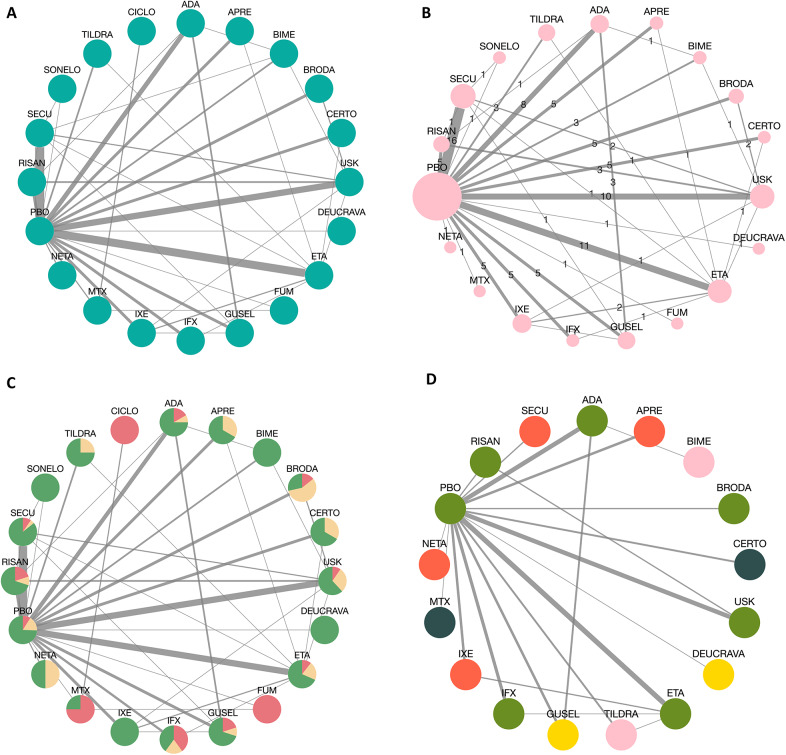
Network graphs for different outcomes. (A) Outcome PASI90: The edge width is proportional to the number of studies for the corresponding comparison. (B) Outcome SAE: Numbers on the edges indicate the number of studies for the corresponding comparisons; node size is proportional to the number of studies that include the corresponding interventions. (C) Outcome AE: Pie charts within the nodes represent the percentage of studies with different risks of bias for the corresponding interventions (red = high, yellow = moderate, green = low). (D) Outcome DLQI: Node colors represent different treatment classes. Figure 1 Long description.

Users can customize the network diagram to display distinct types of information using the provided options. Figure 1 presents network graphs for the four outcomes of our example, each illustrating specific features. In Figure 1A, the network diagram for PASI90 uses edge size to represent the number of trials for each comparison. In Figure 1B, the network diagram for SAE demonstrates additional customization options. Users can also adjust the node size to reflect the number of trials for both interventions and comparisons and include labels on the edges to indicate the number of trials. Node colors can also be customized by specifying a color name or code (e.g., “pink” or “#FFC0CB”). Further, node colors can be modified to represent the risk of bias. For example, in the graph of DLQI (Figure 1C), each node is displayed as a pie chart, showing the proportion of trials with low (green), moderate (yellow), and high (red) risk of bias for the corresponding intervention. Additionally, if treatment class information is included in the data, the diagram can group interventions by color to indicate that they belong to the same treatment class, as shown in Figure 1D for AE.

At the top of the results page, users can choose from a dropdown list to display different outputs. The first option, “Data & Transitivity,” presents data tables, box plots, and scatter plots that are typically used in NMA to evaluate the *transitivity* assumption, which is explained in detail in the Assessing transitivity section. Both raw data and data converted to the contrast-based format are shown prior to the analysis. Additionally, on the setup analysis page, if users upload time-related information such as publication year or study start and end dates, a timeline bar will be displayed on the results page, allowing users to visualize how the evidence has evolved over time.

### 3.4 Assessing transitivity

Before conducting the analysis, it is crucial to assess the validity of the *transitivity* assumption. For an indirect comparison (e.g., A versus B) to be considered valid, it is necessary for the direct comparisons (e.g., A versus C and B versus C) to exhibit similar distributions of key clinical and methodological characteristics (i.e., effect modifiers).^27^ However, substantial empirical evidence suggests that the evaluation of this assumption is often inadequately reported, or not performed, in published NMAs.^11^^,^ ^12^^,^ ^28^

**Figure 2:**
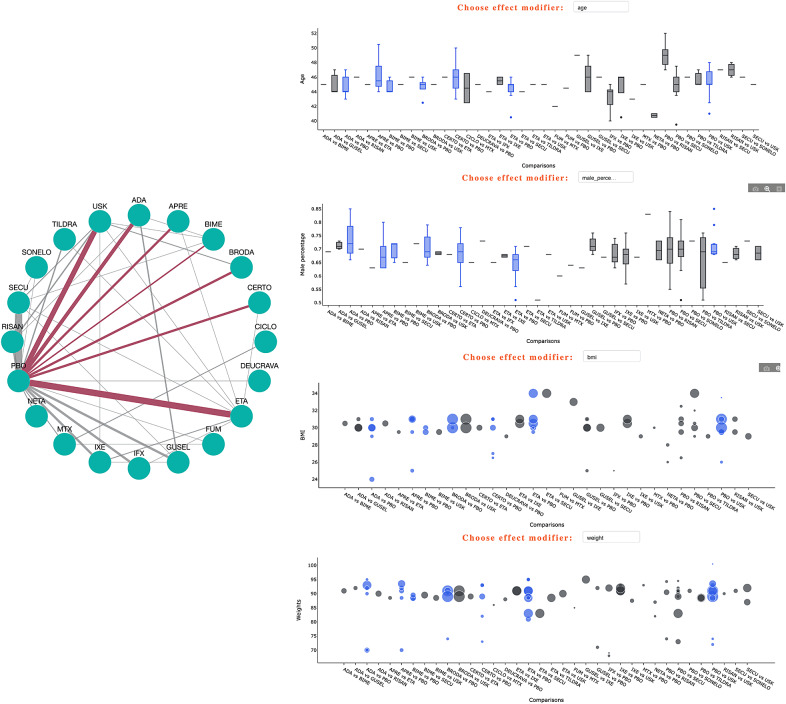
Boxplots of age and male percentage, and scatter plots for BMI and weight for the PASI90 outcome. Seven comparisons (highlighted in blue) are selected by choosing the corresponding edges (shown in red) in the network diagram. Figure 2 Long description.

While the *transitivity* assumption cannot be statistically tested, it can be assessed clinically and epidemiologically.^2^^,^ ^5^^,^ ^29^^,^ ^30^ During the data upload process, users are prompted to select potential effect modifiers from their dataset. Currently, effect modifiers must be in continuous format. For dichotomous variables, users should convert them into proportions (e.g., the percentage of participants with a given characteristic). For categorical variables, users can assign numerical codes to represent categories (e.g., 1, 2, and 3). At the results page, box plots and scatter plots are generated for each selected effect modifier in the dropdown list, allowing users to compare their distribution across the available direct comparisons. In scatter plots, point sizes are proportional to the sample size when that information is provided for each. For example, Figure 2 shows for the psoriasis network box plots for age and percentage of male participants, and scatter plots for BMI and mean weight. Users can highlight specific box plots or scatter plots by selecting the corresponding comparisons in the network diagram. Overall, the distributions of these characteristics appear relatively similar across the comparisons, raising no concerns with respect to *transitivity* for this dataset. However, for some comparisons, the number of available studies is very small (one or two studies only) and no distributions can be obtained.

## 4 Visualizing and exploring results

### 4.1 Forest plots for direct and network summary effects

**Figure 3:**
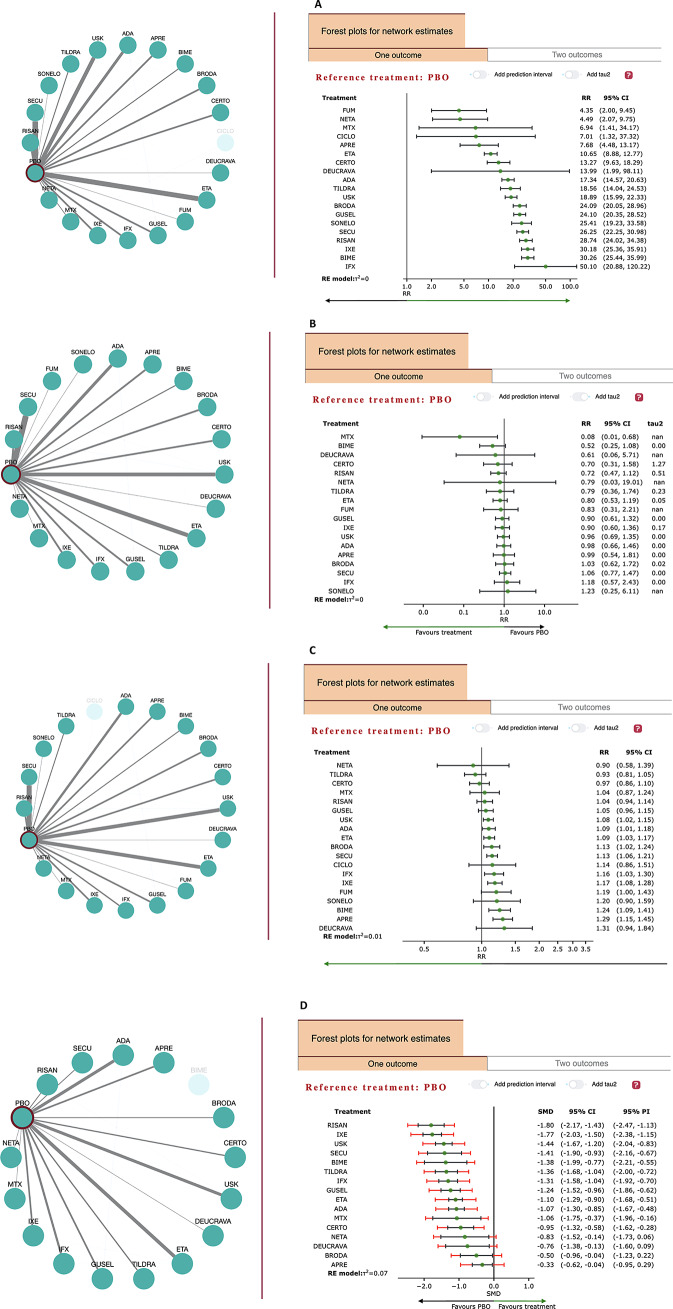
Forest plots for different outcomes with “PBO” as the reference treatment. (A) Outcome PASI90. (B) Outcome SAE. (C) Outcome AE. (D) Outcome DLQI. The black line represents the confidence interval, and the red line represents the prediction interval. CI: Confidence Interval; PI: Prediction Interval; tau2: heterogeneity for each comparison in pairwise meta-analysis. Figure 3 Long description.

The psoriasis example includes 20 distinct treatments, which can provide 20 different NMA forest plots, each with a different reference treatment. In the “Forest plots” tab of NMAstudio 2.0, users can click on any node in the diagram, such as “placebo”, to set it as the reference treatment. This generates the corresponding “standard” NMA forest plot (Figure 3) and a bidimensional forest plot showing the relative effects for two outcomes with the same reference treatment (Supplementary Figure S3) simultaneously. The standard forest plot shows all effect sizes along with confidence intervals, *prediction intervals* (optional), heterogeneity estimates for each comparison (optional), and the common heterogeneity estimate of the NMA, preventing the overemphasis of highly uncertain findings. The bidimensional forest plot enables users to have a more complete view of the results and identify treatments that perform well on two outcomes simultaneously (e.g., efficacy and safety). Given that this graph can become busy and less visible for large networks, users may remove treatments by clicking the respective point in the legend of the graph. For example, in the psoriasis example, we kept the ten most effective treatments using placebo as the reference; those that appear in the upper-right corner are those that perform best both for efficacy and safety (Supplementary Figure S4).

Although summary effects involving both direct and indirect evidence are the main output from NMA, direct evidence and study-level effects also provide important information and need to be easily accessible. For example, high heterogeneity within a certain comparison or the presence of outlying studies may be the cause of increased heterogeneity or inconsistency in the entire network.^30^^,^ ^31^ The psoriasis example includes 58 comparisons with direct evidence, which is challenging to present clearly using traditional formats. NMAstudio 2.0 addresses this problem by allowing users to click on any edge in the diagram, such as the one connecting “PBO” and “ETA” to generate the corresponding pairwise forest plot (Supplementary Figure S5). This feature makes it easy and fast to navigate and review the pairwise forest plots for all or selected direct comparisons.

### 4.2 League tables presenting all NMA relative effects

**Figure 4:**
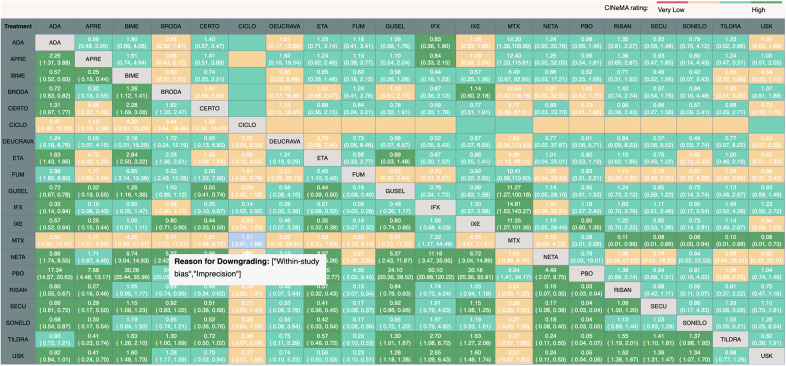
League table with color-coded CINeMA ratings. The upper triangle represents PASI90, and the lower triangle represents SAE. Hovering over the cell comparing MTX and BIME displays the reasons for downgrading. Figure 4 Long description.

League tables typically display the summary relative effects along with the respective confidence intervals for every treatment contrast in the network. They often present the results of two outcomes, one in the upper triangle and the second in the lower triangle. In NMAstudio 2.0, two types of league tables are offered. The first is for a single outcome, with the upper triangle showing direct estimates and the lower triangle showing mixed (NMA) estimates. The second is for two outcomes, where both the upper and lower triangles display the mixed effects for each outcome. Users can enhance league tables by coloring the cells based on either the overall risk of bias of the respective contrast or the overall confidence rating. The latter is obtained using the *CINeMA* framework;^32^ users need first to upload the final report as obtained from the *CINeMA* software (cinema.med.auth.gr). Users can also hover over the colored cells of the table to see reasons for downgrading the evidence as these appear in the *CINeMA* report (Figure 4). This makes it straightforward to identify interventions and comparisons with higher and lower confidence in the evidence. The league table is also connected to the network diagram, allowing users to select specific interventions in the diagram and display the relevant comparisons in the table in the desired order (Supplementary Figure S6).

### 4.3 Ranking of the interventions

NMAstudio 2.0 generates two ranking visualizations: a *P-score* heatmap for all the outcomes analyzed and a two-dimensional scatter plot displaying the *P-scores* for two selected outcomes (Supplementary Figures S7 and S8). *P-scores* rank treatments based on all relative effects in the league table while also considering their precision, reflecting the average certainty that a treatment is better than its competitors.^33^ Consideration of ranking results for different outcomes jointly is crucial for proper decision-making. In the two-dimensional scatter plot, the size of the points is proportional to the number of studies that include the corresponding interventions, and the different colors represent different clusters of interventions with similar performance based on the p-score values. In our example, IFX demonstrates the highest score for the efficacy outcome (PASI90) but has a notably low *P-score* for the safety outcome (SAE). Also, ranking should always be considered along with the NMA relative effects and other characteristics, such as risk of bias or amount of evidence.^34^ Examining the ranking alongside the network diagram, with risk of bias indicated by color and node size reflecting the number of trials for each intervention (Supplementary Figure S9), reveals that IFX has substantial information coming from low risk of bias studies, while it is mainly compared with placebo, and only one study connects it directly with ETA. The two-dimensional scatter plot suggests that BIME might be the best choice, as it is positioned in the top-right corner of the graph (Supplementary Figure S8).

## 5 Consistency and reporting bias

*Consistency* refers to the statistical agreement between direct and indirect evidence for a given comparison.^2^^,^ ^5^ There are two types of tests for inconsistency: local and global tests. A local test can only be performed when a certain intervention comparison is included in one or more closed loops in the network.^35^ If direct and indirect evidence are inconsistent, there are concerns with respect to the validity of the NMA results.^2^^,^ ^30^ NMAstudio 2.0 offers two tests for inconsistency: the side-splitting approach that evaluates inconsistency for every comparison informed both directly and indirectly^36^ and the design-by-treatment interaction model that provides a global test for the entire network.^37^ For the outcome PASI90 of the psoriasis example, the global test indicates no significant inconsistency. However, the local test highlights potential issues for the IXE versus USK comparison (p-value = 0.04), suggesting that combining direct and indirect effects for this comparison might be problematic and requires further investigation.^2^^,^ ^5^ The local consistency results table is also connected to the network diagram, allowing users to select specific comparisons displayed in the table (Supplementary Figure S10). Here, the suspicious comparison is only informed by one study, which might explain the difference between direct and indirect effects.

Reporting bias poses a significant threat to the validity of results in systematic reviews. Small-study effects are a common issue and refer to the systematic differences in the results between smaller and larger studies.^38^ Small-study effects can be visually assessed using funnel plots, where asymmetry may indicate the presence of small-study effects and requires further investigation.^39^ NMAstudio 2.0 provides *comparison-adjusted funnel plots* to examine small-study effects across the entire network^40^ and standard funnel plots for specific comparisons. For comparison-adjusted funnel plots, users should select one treatment in the network diagram to be used as a reference for all comparisons. This prevents misleading interpretations that may arise when there is not a consistent direction for all comparisons in the graph. In the psoriasis example, we chose PBO as the reference treatment; in this way, the produced funnel plot assesses whether smaller studies tend to favor active interventions against PBO more than larger studies do. For the four outcomes of the psoriasis network, the funnel plots appear symmetrical, raising no concerns with respect to the presence of small-study effects (Supplementary Figures S11–S14). For standard funnel plots, users can select a comparison that includes at least two studies in the network diagram to generate the plot. However, to avoid misleading interpretations, asymmetry should be judged only for comparisons with at least ten studies. For example, Supplementary Figure S15 presents the standard funnel plot for the comparison “PBO versus ETA” for the PASI90 outcome, showing no visible asymmetry, which suggests no evidence of small-study effects for this comparison.

## 6 Data sharing and open science

Transparency, openness, and reproducibility are essential features of science that should be standard practice.^13^ In a typical NMA, authors present key findings in the main text while additional results, raw data, code, and other materials are placed in the supplementary material. However, the supplementary material often receives less attention due to its large volume, requiring substantial time for thorough exploration. The fixed format of supplementary material also limits readers’ flexibility in exploring results. These challenges hinder the efficient sharing of data and results and prevent readers from deeply delving into the findings.

To promote an open research culture, NMAstudio 2.0 provides easy functions for saving and loading uploaded data, allowing users to store and share their projects. On the results page, users can click the *Save/Load Project* button to generate a unique token for data sharing (Supplementary Figure S16). This token can be shared by authors alongside their published article. Readers, peer-reviewers, or other stakeholders can then use the “Load Project” function in NMAstudio 2.0 to enter the token, reload all results of the previously saved project, and explore the results interactively (Supplementary Figure S17). With its interactive and user-friendly features, NMAstudio 2.0 gives access to original data and results, supporting independent verification and offering reviewers flexibility to explore findings and draw evidence-based conclusions. Of course, this feature is not intended to replace traditional supplementary materials entirely, but rather to complement them by offering an interactive and user-friendly way to explore data, results, and assumptions.

## 7 Discussion

In this paper, we introduce NMAstudio 2.0, a web application that aims to facilitate the production, visualization, and communication of NMAs. Its underlying motivation is to continuously address existing and new challenges that most investigators encounter when conducting or using NMAs. We illustrate how NMAstudio 2.0 can simplify the entire analytical process and enhance the interpretation and understanding of NMA findings using a Cochrane Review that involves data on multiple treatments and multiple outcomes.^23^ NMAstudio’s 2.0 unique design combines a user-friendly “point-and-click” environment with extensive interactivity and a direct connection between the network diagram and all outputs. Other web-based applications for NMA are also available.^41^^–^ ^44^ However, to our knowledge, NMAstudio 2.0 is the only tool, to date, that allows the analysis of multiple outcomes simultaneously, visualizing the evolution of evidence over time, evaluating *transitivity*, facilitating data sharing, and offering such a high level of interaction with data and results. Moreover, NMAstudio 2.0 allows flexibility in the preparation of the data file with no restrictions on variable names or the number of study arms. Users benefit from a wide range of interactive, high-quality plots that are unique to NMAstudio 2.0 and not derived from other packages. These plots are generated in a single analysis run, making the process efficient, fast, and user-friendly.

A usual concern for tools that aim to simplify NMAs and statistical analyses, in general, is the risk of an increased number of poorly conducted studies and invalid results. NMAstudio 2.0 involves steps during the data upload process that aim to mitigate, to some degree, this risk. First, users are asked to provide the link to their protocol to prevent data-driven decisions. Second, they need to upload risk of bias information for the included studies, which then appears automatically in the league table. Third, users are asked to upload data on the potential effect modifiers, which are used for the evaluation of *transitivity*. If any of these steps are skipped, warnings appear on the results page to inform end-users about the omission of these steps and the associated risks. Despite these steps, it is essential to emphasize, though, that involvement of statisticians and methodologists in NMA projects remains indispensable. One limitation of the current version of the tool is that it does not allow viewing the actual R functions executed during analysis. Additionally, software sustainability is an important consideration. It refers to the capacity of a software tool to remain reliable, maintainable, and accessible over time, independent of changes in technology or the duration of specific funding sources. NMAstudio 2.0 will address this by offering self-hosting options, which allow users to run the software independently of external servers, and by ensuring that users retain ownership of their data.

Further updates are under development. NMAstudio 2.0 currently offers only the conduct of NMA in the frequentist framework using the netmeta package in R^21^ and the inverse-variance model. Additional models available in netmeta, such as the Mantel–Haenszel or the penalized likelihood models,^45^^,^ ^46^ as well as Bayesian NMA, will be implemented in upcoming versions of the application. We are also working on allowing the conduct of sensitivity and subgroup analyses based on pre-defined (during data uploading) study and intervention characteristics. Comparison of results between primary and exploratory analyses will be enhanced through the interactive features that NMAstudio 2.0 offers. Additionally, NMAstudio 2.0 will allow for a full order of the treatments in the comparison-adjusted funnel plots. Finally, we plan to allow users to upload NMA results obtained from external analyses and use NMAstudio 2.0 for visualization purposes. To further enhance the comprehensiveness and interpretation of NMA findings for end-users, a knowledge translation tool is being developed for integration into NMAstudio 2.0. This tool is designed to provide an interactive, flexible, and user-friendly approach to summarizing and presenting NMA results within a single page. By featuring two versions—a standard version and an advanced version—it aims to improve the accessibility and usability of NMA findings for a broad range of stakeholders.

## 8 Conclusions

We believe NMAstudio 2.0 serves as a valuable resource for researchers and clinical practitioners embarking on an NMA. Its unique interactive features facilitate the communication of NMA outputs and enhance the clarity of results for diverse audiences. At the same time, it offers increased flexibility and interactivity, while prompting users to adhere to high standards for conducting and reporting NMAs. Future versions of NMAstudio 2.0, together with the upcoming knowledge translation tool, have the potential to further promote transparency, reproducibility, data sharing, open science, and broad research collaboration.

## Supporting information

10.1017/rsm.2026.10074.sm001 Yu et al. supplementary material 1 Yu et al. supplementary material

10.1017/rsm.2026.10074.sm002 Yu et al. supplementary material 2 Yu et al. supplementary material

10.1017/rsm.2026.10074.sm003 Yu et al. supplementary material 3 Yu et al. supplementary material

## Data Availability

All data and code can be obtained via https://doi.org/10.5281/zenodo.18368497 or https://github.com/CER-METHODS/NMAstudio-2.0

## Supplementary material

To view supplementary material for this article, please visit http://doi.org/10.1017/rsm.2026.10074.

## Long descriptions

### Figure 1 Long description

A multi-panel figure containing four circular network graphs. Each graph features nodes representing treatments arranged in a circle, with lines connecting them to indicate direct comparisons.

Panel A, Outcome P A S I 90. All nodes are uniform teal circles. The thickest edges connect P B O to E T A, S E C U, and I X E. Other nodes include A D A, A P R E, B I M E, B R O D A, C E R T O, U S K, D E U C R A V A, F U M, G U S E L, I F X, M T X, N E T A, R I S A N, S O N E L O, T I L D R A, and C I C L O.

Panel B, Outcome S A E. Nodes are light pink. The P B O node is significantly larger than others. Edges include numerical labels indicating study counts. The highest count is 16 between P B O and R I S A N, followed by 11 between P B O and E T A, and 10 between P B O and U S K.

Panel C, Outcome A E. Nodes are pie charts showing risk of bias. Green represents low risk, yellow moderate, and red high. C I C L O and F U M are entirely red. A D A, G U S E L, and I X E are predominantly green. P B O shows a mix of all three colors.

Panel D, Outcome D L Q I. Nodes are color-coded by treatment class. A D A, R I S A N, P B O, B R O D A, U S K, E T A, G U S E L, and I F X are olive green. S E C U, A P R E, N E T A, and I X E are orange. M T X and C E R T O are dark slate gray. B I M E and T I L D R A are light pink. D E U C R A V A and G U S E L are yellow.

Navigate back to Figure 1.

### Figure 2 Long description

The figure is divided into two main sections. On the left is a circular network diagram with teal nodes representing treatments such as A D A, A P R E, B I M E, and P B O. Red lines highlight specific connections originating from P B O to other nodes. On the right, four plots are stacked vertically, each sharing an x-axis labeled Comparisons with treatment pairs like A D A vs B I M E.

* The top plot is a boxplot for Age, ranging from 40 to 52. Blue boxes highlight the seven selected comparisons.

* The second plot is a boxplot for Male percentage, ranging from 0.5 to 0.85, with blue boxes indicating the same selected comparisons.

* The third plot is a scatter plot for B M I, ranging from 24 to 34. Data points are represented by circles of varying sizes, with blue circles highlighting the selected comparisons against grey background data.

* The bottom plot is a scatter plot for Weights, ranging from 70 to 100. Similar to the B M I plot, blue circles highlight the specific comparisons selected in the network diagram.

Navigate back to Figure 2.

### Figure 3 Long description

A four-panel multi-plot labeled A through D. Each panel contains a circular network diagram on the left and a corresponding forest plot on the right.

Panel A: Outcome P A S I 90. The network diagram shows P B O connected to 18 treatments including U S K, A D A, and I F X. The forest plot lists treatments from F U M at the top to I F X at the bottom. Relative Risk R R values range from 4.35 to 50.10, showing all treatments are more effective than P B O for this outcome.

Panel B: Outcome S A E. The forest plot shows R R values centered around 1.0, indicating no significant difference in Serious Adverse Events between treatments like M T X, B I M E, and P B O. The x-axis is labeled Favours treatment to the left and Favours P B O to the right.

Panel C: Outcome A E. Similar to panel B, the forest plot for Adverse Events shows most treatments clustered near the vertical line of no effect at R R 1.0, with N E T A at 0.90 and D E U C R A V A at 1.31.

Panel D: Outcome D L Q I. This panel uses Standardized Mean Difference S M D. The forest plot includes red prediction interval lines and black confidence interval lines. Values range from R I S A N at negative 1.80 to A P R E at negative 0.33. All data points fall to the left of the zero line, indicating they favour treatment over P B O for quality of life improvement.

Common elements across all panels include a 95 percent C I column and a reference to the R E model tau-squared heterogeneity value at the bottom left of each forest plot.

Navigate back to Figure 3.

### Figure 4 Long description

A square matrix grid displays treatment comparisons. The top horizontal axis and left vertical axis list treatment initialisms including A D A, A P R E, B I M E, B R O D A, C E R T O, C I C L O, D E U C R A V A, E T A, F U M, G U S E L, I F X, I X E, M T X, N E T A, P B O, R I S A N, S E C U, S O N E L O, T I L D R A, and U S K.

At the top right, a legend for C I N e M A ratings shows a color gradient from dark orange for Very Low to dark green for High. The diagonal cells of the matrix are grey and contain the treatment names.

The matrix is divided into two triangular sections. The upper right triangle represents P A S I 90 data, and the lower left triangle represents S A E data. Each cell contains a numerical value and a confidence interval in brackets.

A white pop-up box is visible, triggered by hovering over the intersection of M T X and B I M E. The box is titled Reason for Downgrading and lists Within-study bias and Imprecision as the factors for the rating. The cell being hovered over is highlighted with a blue border and contains the value 1.01 with a confidence interval of 0.10 to 10.08.

Navigate back to Figure 4.
