# Supplementary material for: NMAstudio 2.0: An interactive tool for network meta-analysis to enhance understanding, interpretation, and communication of the findings
Source: Res Synth Methods. 2026 Mar 6;17(4):836–49. doi: 10.1017/rsm.2026.10074 (PMC13311350; doi:10.1017/rsm.2026.10074)

Step 1. Provide the link of the protocol

Provide the link of the protocol of your study:

https://www.cochranelibrary OK Skip

*NMAstudio requires users to provide protocol links before running analysis.

Step 2. Upload the dataset

Drag and Drop or Select a File

psoriasis_4_outcomes.csv

*The dataset should be uploaded as the csv format. Missing values should be encoded as '.', 'NA' or empty cells in the uploaded dataset.

Step 3. Select the format of the dataset

Select the format of your dataset:

- (•) long
- ( ) wide
- ( ) iv

*You can click the link to see the differences between the formats:

Link

Step 4. Select the overall variables

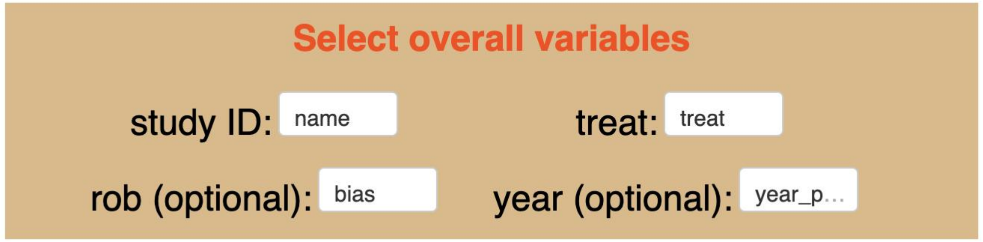

*study ID: study ID or study name

*rob: risk of bias should be encoded in your data file as either {1,2,3}, {l,m,h} or {L,M,H}, the arms in the same study should have the same rob value.

*year: year of publication

Step 5. Enter the number of outcomes

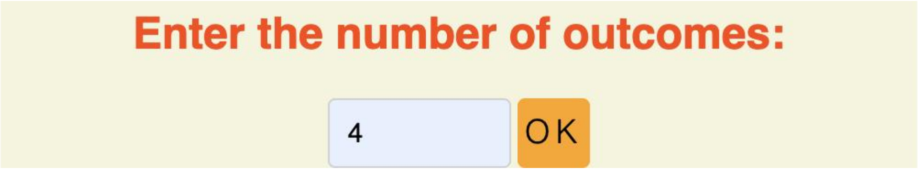

*NMAstudio now supports any number of outcomes.

Step 6. Select two outcomes for league table

Select two outcomes for league table

- [x] outcome 1
- [x] outcome 2
- [ ] outcome 3
- [ ] **Skip**
- [ ] outcome 4

*Select two primary outcomes for league table.
Select "Skip" if there is only one outcome.

Step 7. Select the type of each outcome

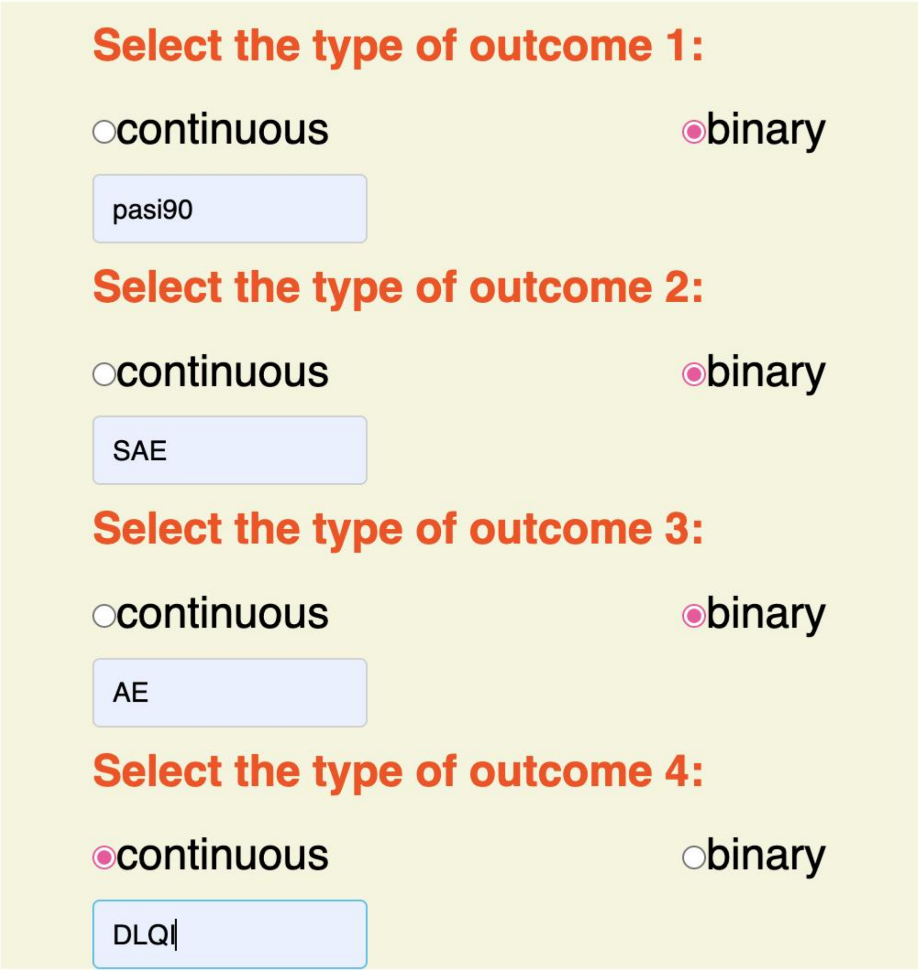

*Select binary or continuous and enter the corresponding name for each outcome.

Step 8. Fill other variables for each outcome

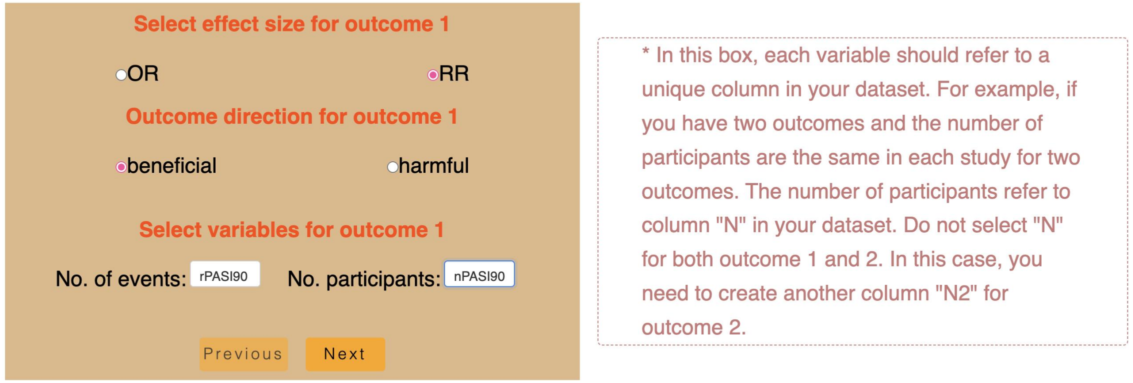

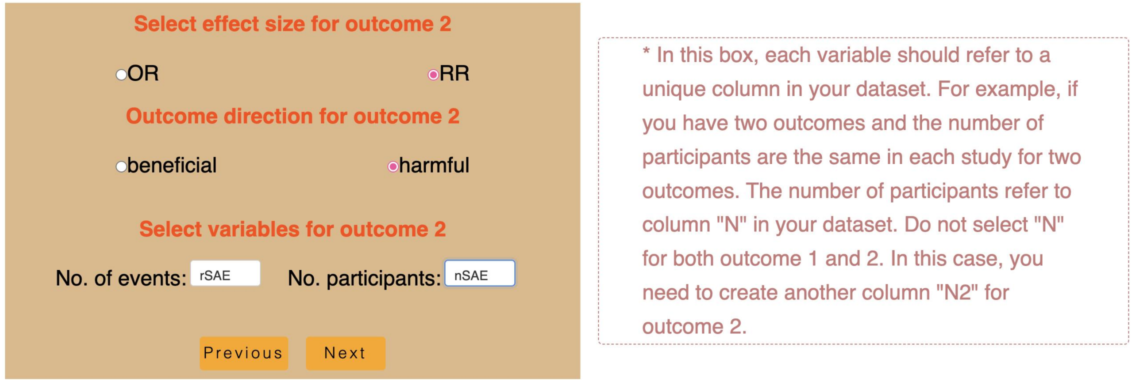

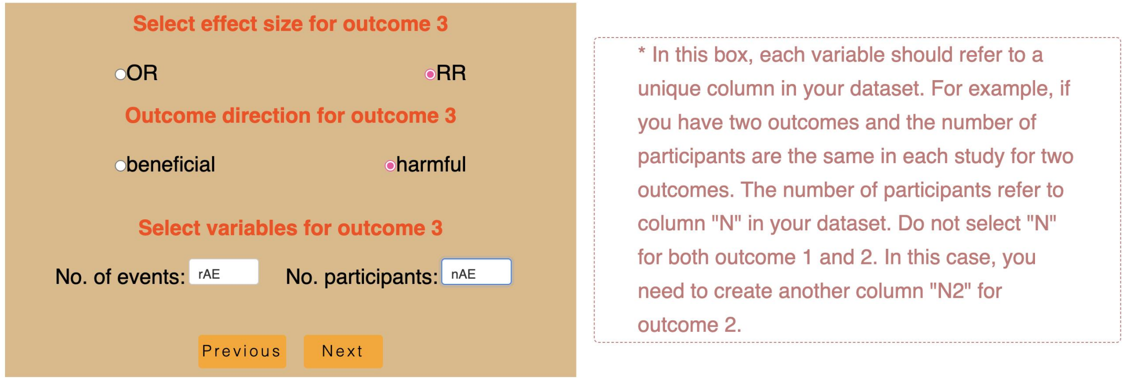

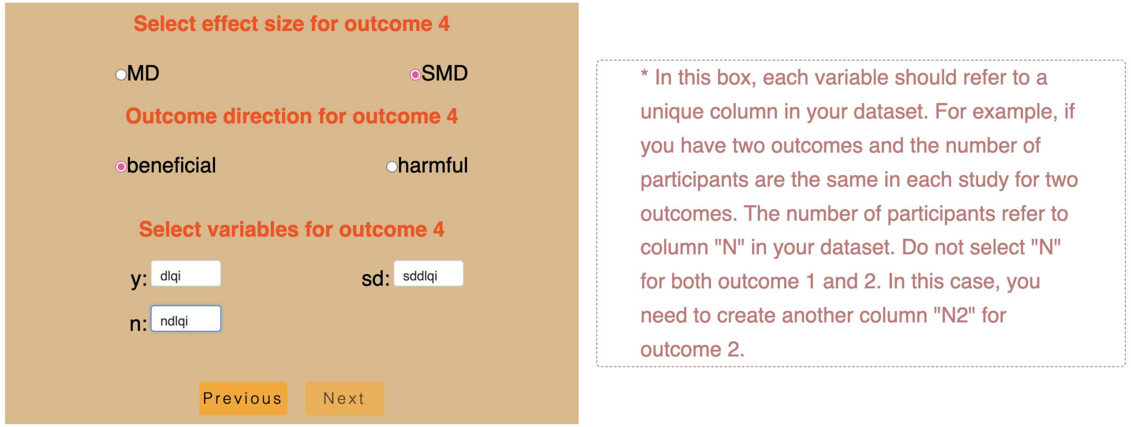

Step 9. Select potential effect modifiers

Select potential effect modifiers

[ ] unique_id	[ ] name	[ ] bias
[ ] year_publication	[ ] treat	[ ] treat_class
[ ] nPASI90	[ ] rPASI90	[ ] rAE
[ ] nAE	[ ] rSAE	[ ] nSAE
[ ] dlqi	[ ] sddlqi	[ ] ndlqi
[x] age	[x] male_percentage	[x] weight
[x] bmi		

[ ] **Skip**

*Select potential effect modifiers you want to check. If you do not want to check, please tick "Skip".
*When you upload long format dataset, the arms in the same study should have the same effect modifier value.

Step 10. Click to run analysis

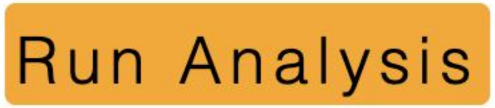

Supplement: Yu et al. supplementary material 1 — Yu et al. supplementary material [file S175928792610074Xsup001.pdf]
