# Supplementary material for: NMAstudio 2.0: An interactive tool for network meta-analysis to enhance understanding, interpretation, and communication of the findings
Source: Res Synth Methods. 2026 Mar 6;17(4):836–49. doi: 10.1017/rsm.2026.10074 (PMC13311350; doi:10.1017/rsm.2026.10074)
Supplement: Yu et al. supplementary material 3 — Yu et al. supplementary material [file S175928792610074Xsup003.docx]

Supplementary Material

[Homepage 2](#_Toc208844140)

[Setup Analysis Page 3](#_Toc208844141)

[Results Page 4](#_Toc208844142)

[Full names and abbreviations of the treatments 5](#_Toc208844143)

[Three dataset formats 6](#_Toc208844144)

[Figure S1. Warnings in the results page 7](#_Toc208844145)

[Figure S2. Running analysis window 8](#_Toc208844147)

[Figure S3. The bi-dimentional forestplot for outcome PASI90 and SAE when “PBO” as reference treatment 9](#_Toc208844148)

[Figure S4. The bi-dimentional forestplot with the ten most effective treatments using placebo as reference for outcome PASI90 and SAE when “PBO” as reference treatment 10](#_Toc208844149)

[Figure S5. The pairwise forestplot of ETA vs PBO for outcome PASI90. 11](#_Toc208844150)

[Figure S6. The league table of selected interventions for the outcome PASI90 12](#_Toc208844151)

[Figure S7. Ranking heatmaps for four outcomes, with values in the plots representing the P-score 13](#_Toc208844152)

[Figure S8. The p-score scatter plot for outcome PASI90 and SAE 14](#_Toc208844153)

[Figure S9. The network diagram for IFX selection, along with the ranking heatmaps 15](#_Toc208844154)

[Figure S10. The consistency check results table, filtered by the selected edges within the diagram for the PASI90 outcome 16](#_Toc208844156)

[Figure S11. The comparison-adjusted funnel plot for the PASI90 outcome 17](#_Toc208844157)

[Figure S12. The comparison-adjusted funnel plot for the SAE outcome 18](#_Toc208844158)

[Figure S13. The comparison-adjusted funnel plot for the AE outcome 19](#_Toc208844159)

[Figure S14. The comparison-adjusted funnel plot for the DLQI outcome 20](#_Toc208844160)

[Figure S15. The standard funnel plot of comparison ‘PBO VS ETA’ for the PASI90 outcome 21](#_Toc208844161)

[Figure S16. Save the project 22](#_Toc208844162)

[Figure S17. Load the project 23](#_Toc208844163)

# Homepage

# Setup Analysis Page
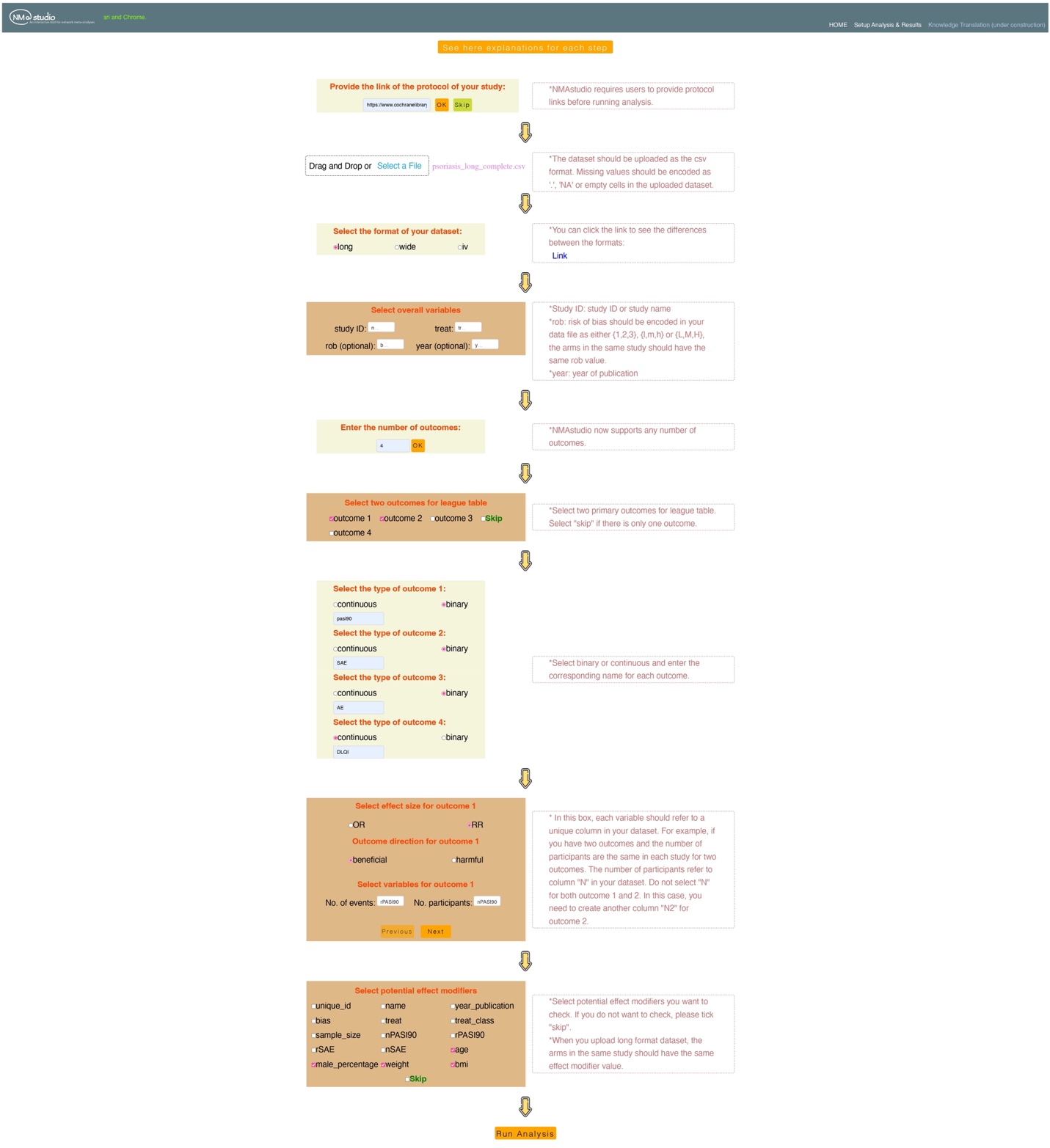


# Results Page


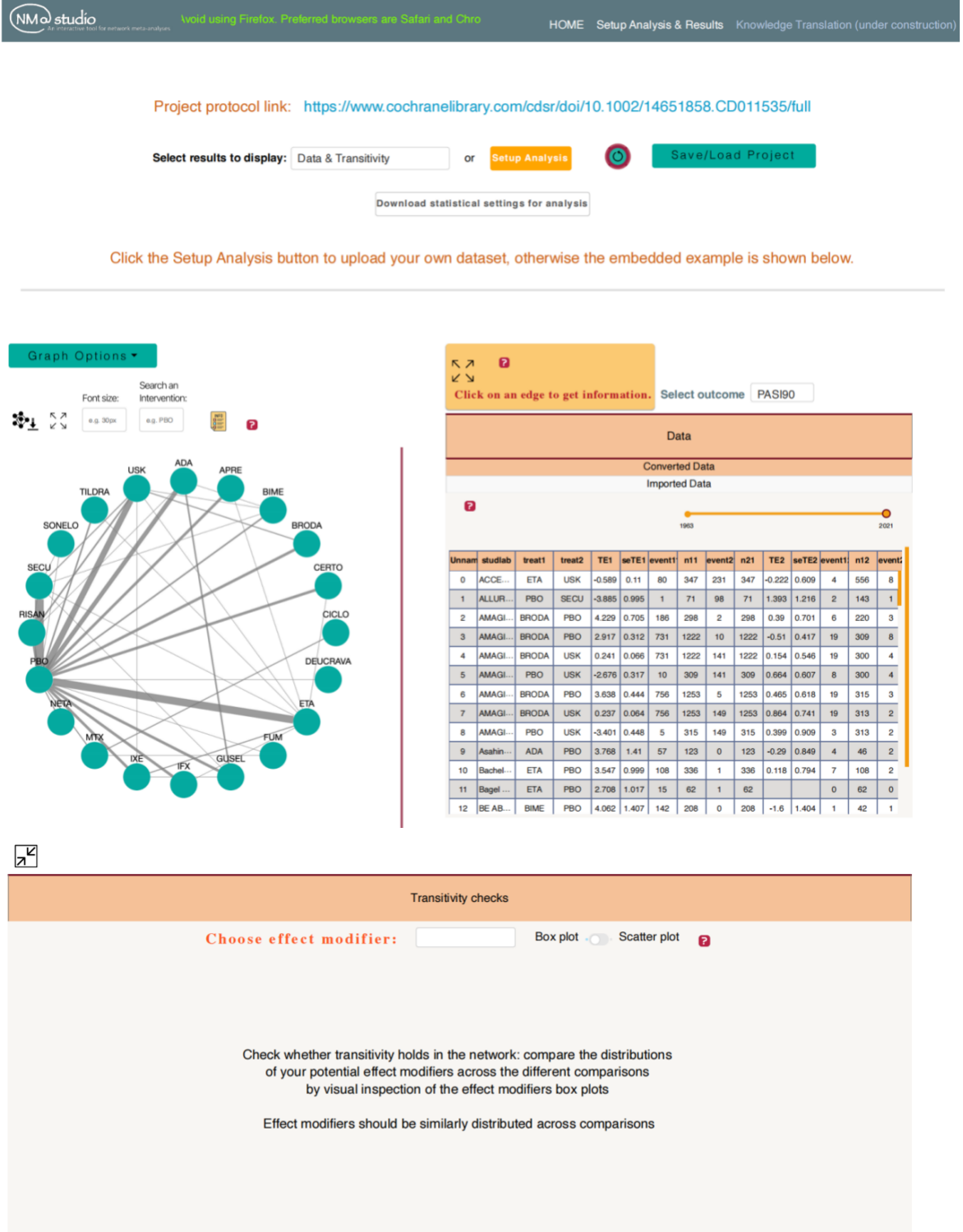


# Full names and abbreviations of the treatments

| **Abbreviation** | **Treatment** |
| --- | --- |
| ADA | Adalimumab |
| APRE | Apremilast |
| BIME | Bimekizumab |
| BRODA | Brodalumab |
| CERTO | Certolizumab |
| CICLO | Ciclosporin |
| DEUCRAVA | Deucravacitinib |
| ETA | Etanercept |
| FUM | Fumaric Acid |
| IFX | Infliximab |
| IXE | Ixekizumab |
| GUSEL | Guselkumab |
| MTX | Methotrexate |
| NETA | Netakimab |
| PBO | Placebo |
| RISAN | Risankizumab |
| SECU | Secukinumab |
| SONELO | Sonelokimab |
| TILDRA | Tildrakizumab |
| USK | Ustekinumab |

# Three dataset formats

**Long format**

| StudyID | Intervention | r | n |
| --- | --- | --- | --- |
| 1 | treatment A | 10 | 50 |
| 1 | treatment B | 20 | 60 |
| 2 | treatment A | 11 | 70 |
| 2 | treatment B | 30 | 75 |
| 2 | Placebo | 5 | 80 |

**Wide format**

| StudyID | Intervention 1 | Intervention 2 | r for intervention1 | r for intervention2 | n for intervention1 | n for intervention2 |
| --- | --- | --- | --- | --- | --- | --- |
| 1 | treatment A | treatment B | 10 | 20 | 50 | 60 |
| 2 | treatment A | treatment B | 11 | 30 | 70 | 75 |
| 2 | treatment A | Placebo | 11 | 5 | 70 | 80 |
| 2 | treatment B | Placebo | 30 | 5 | 75 | 80 |

**Contrast format**

| StudyID | Intervention 1 | Intervention 2 | TE | seTE |
| --- | --- | --- | --- | --- |
| 1 | treatment A | treatment B | 10 | 20 |
| 2 | treatment A | treatment B | 11 | 30 |
| 2 | treatment A | Placebo | 11 | 5 |
| 2 | treatment B | Placebo | 30 | 5 |

# **
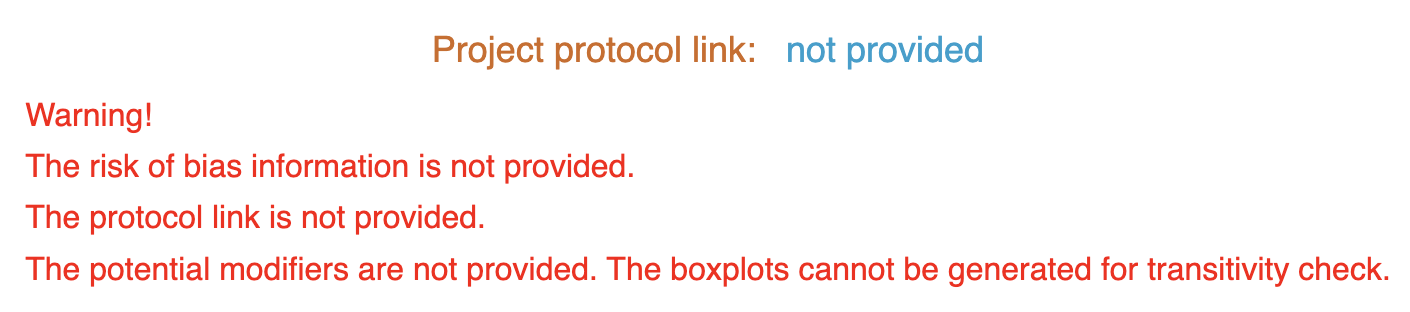
Figure S1.** Warnings in the results page

# **
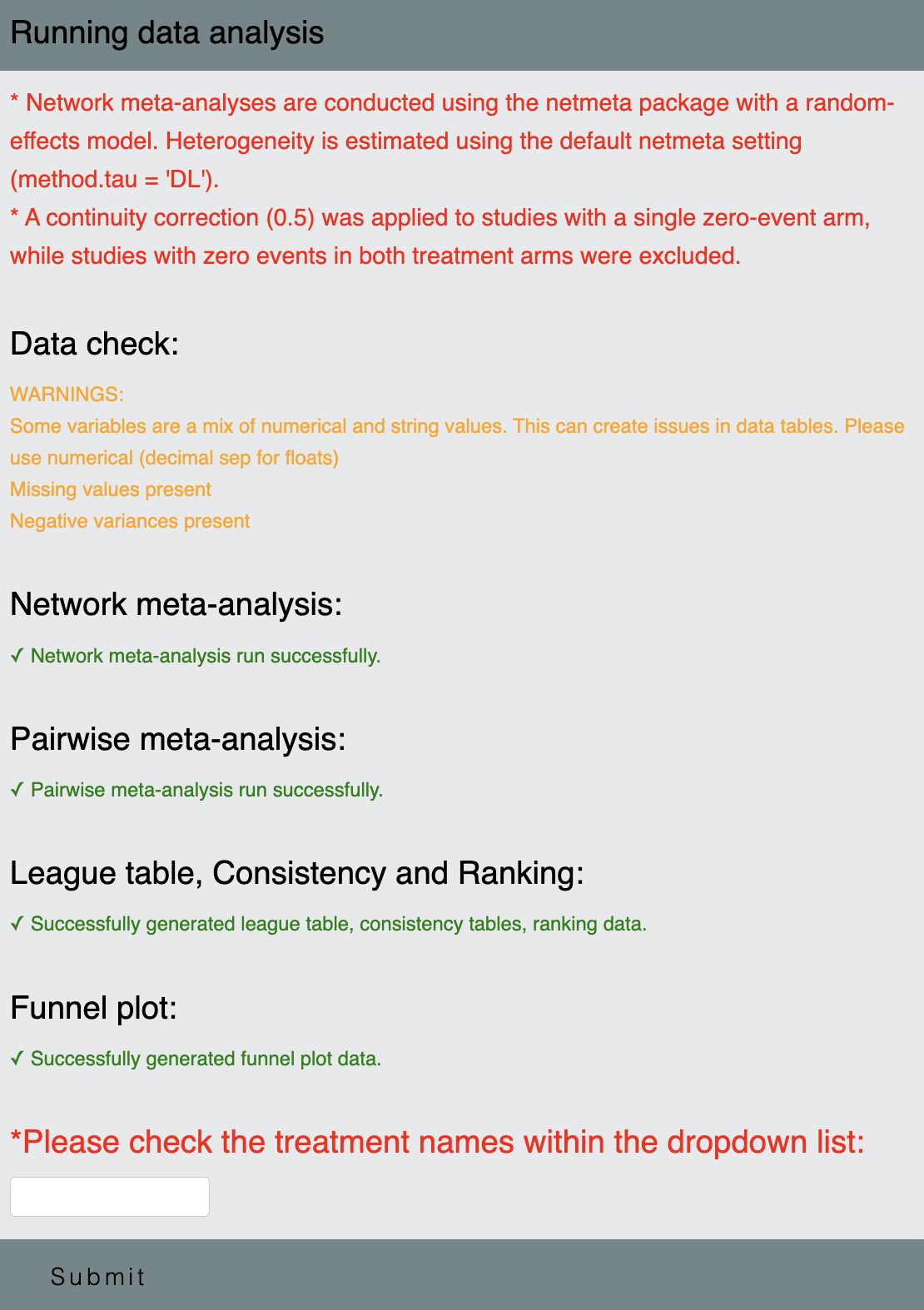
**

# **Figure S2.** Running analysis window

During data analysis, additional warnings may appear in the “data check” step. These warnings point to issues in the dataset rather than validation steps. For example, a warning such as “Some variables are a mix of numerical and string values” indicates inconsistent formatting (e.g., both 32 and "32" in the same column). This may affect some visualizations (like boxplots) but not the NMA itself. Similarly, warnings may indicate missing values in the dataset, which do not stop the NMA from running. However, if the dataset issues affect the NMA process, the analysis will stop and the related errors will be displayed.


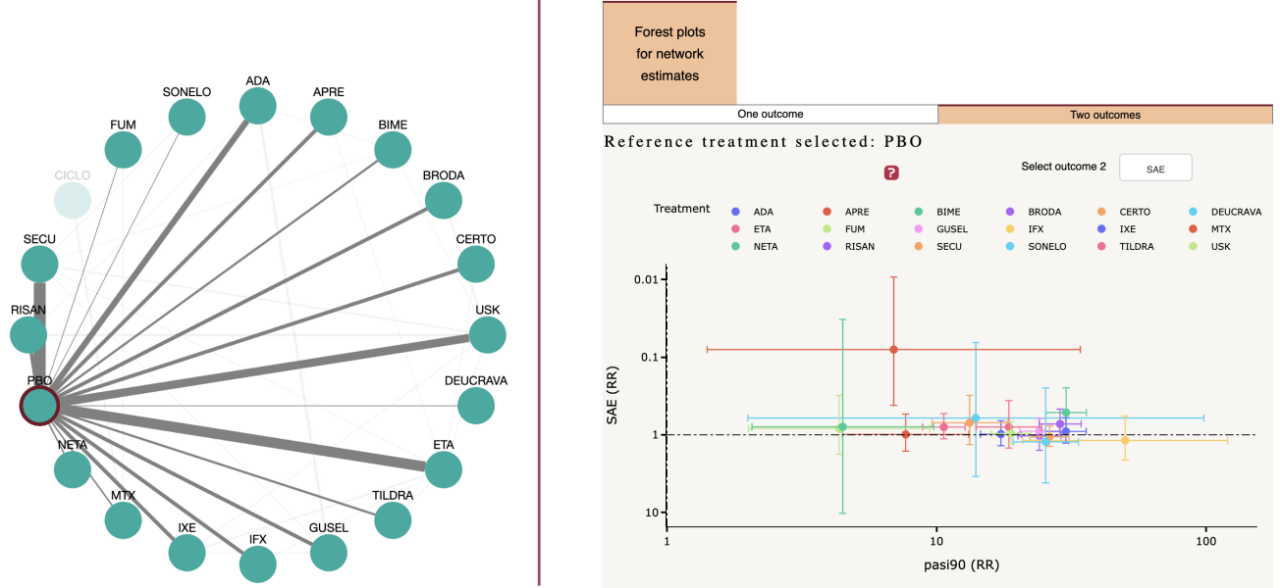


# **Figure S3.** The bi-dimensional forestplot for outcome PASI90 and SAE when “PBO” as reference treatment. Clicking on the reference intervention within the diagram generates the forestplot.


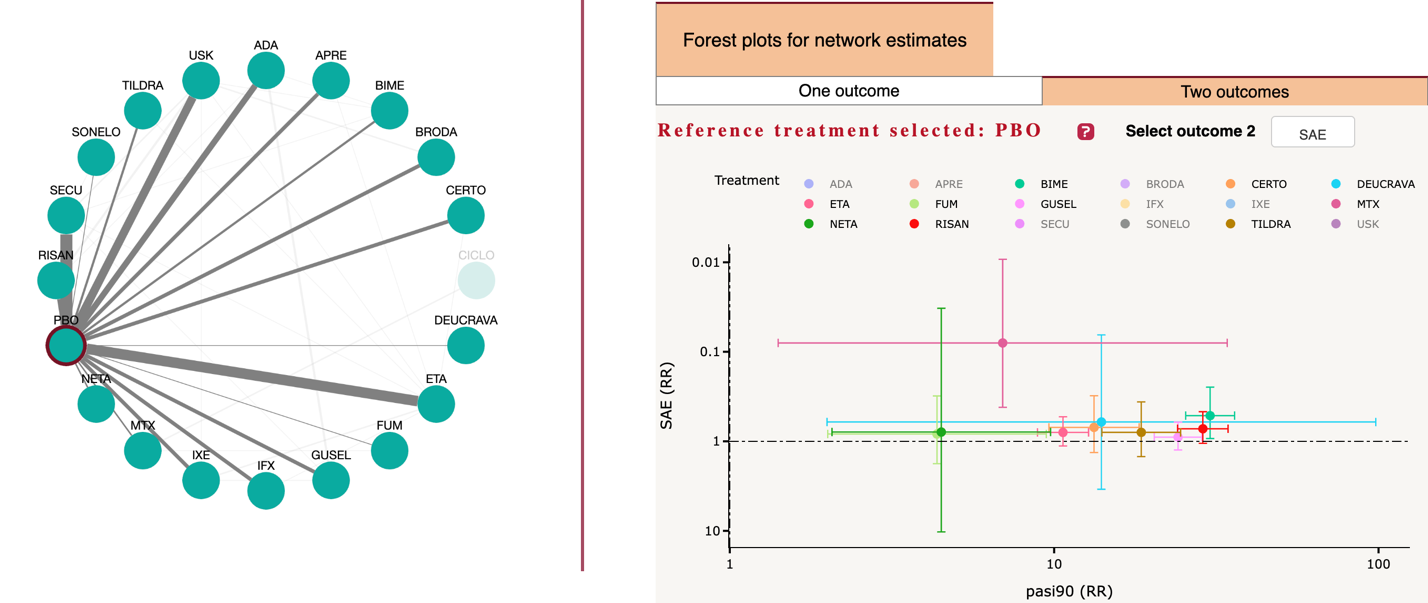


# **Figure S4.** The bi-dimensional forestplot with the ten most effective treatments using placebo as reference for outcome PASI90 and SAE when “PBO” as reference treatment. Remove treatments by clicking the respective point in the legend of the graph.


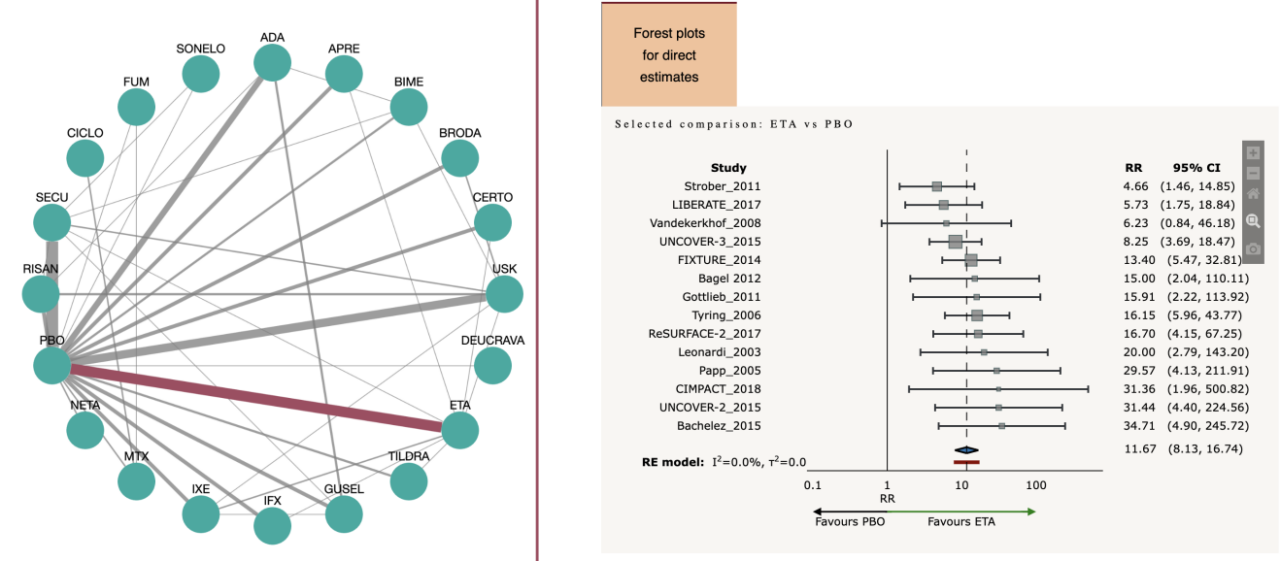


# **Figure S5.** The pairwise forestplot of ETA vs PBO for outcome PASI90. Clicking on the comparison within the diagram generates the forestplot.

#
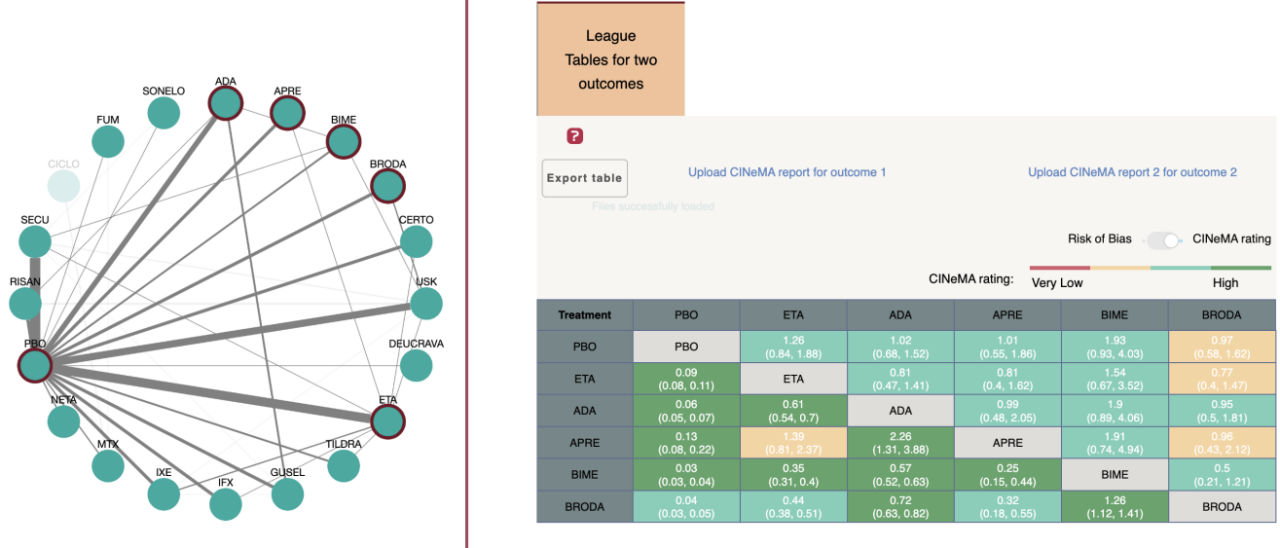
**Figure S6.** The league table of selected interventions for the outcome PASI90. The table can be filtered by selecting the nodes within the diagram, which updates the table to display only the corresponding interventions.


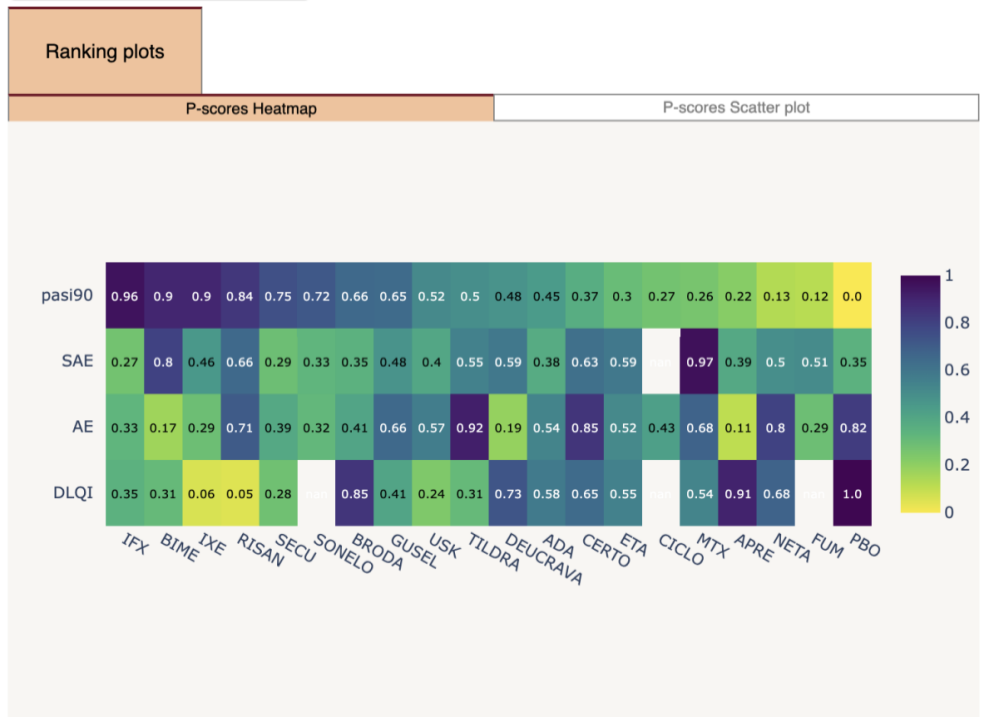


# **Figure S7.** Ranking heatmaps for four outcomes, with values in the plots representing the P-score.


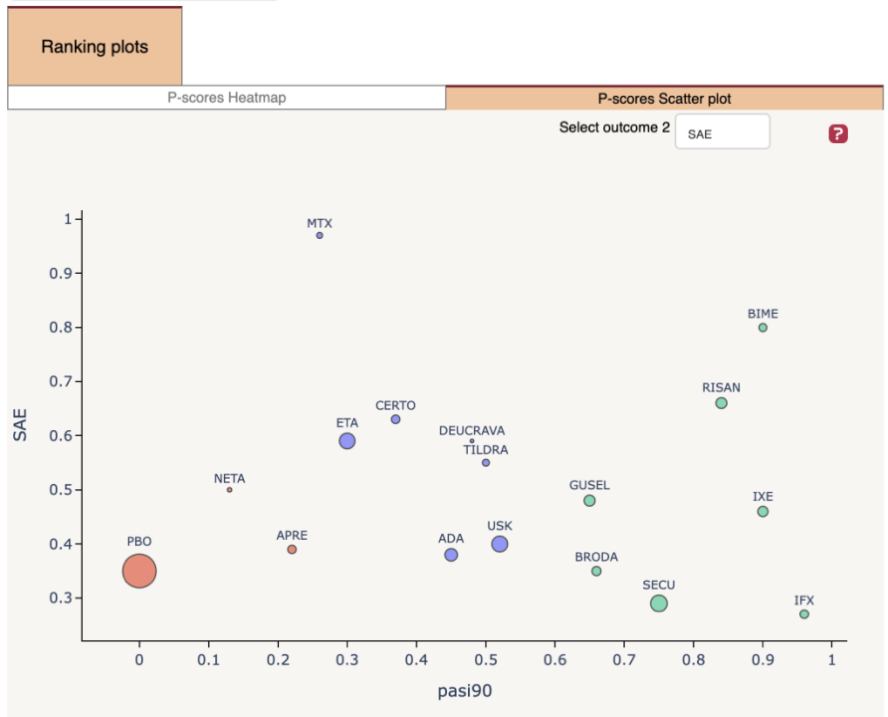


# **Figure S8.** The p-score scatter plot for outcome PASI90 and SAE.

**
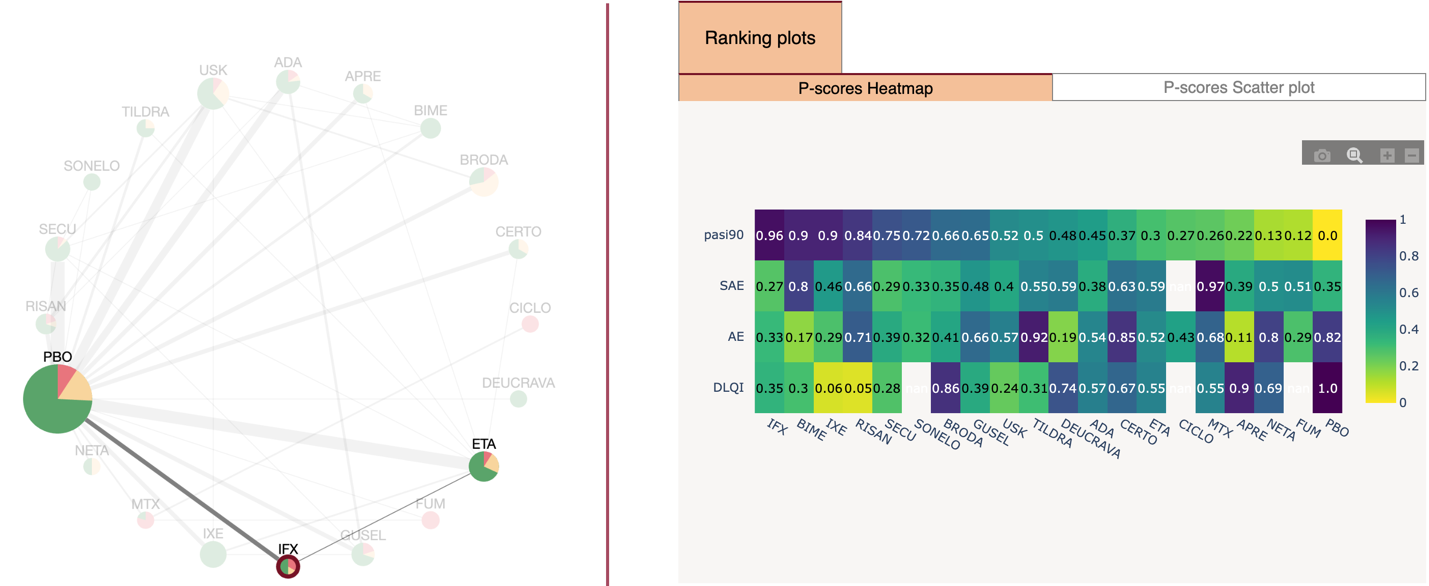
**

# **Figure S9.** The network diagram with the risk of bias colors and the node size reflecting the number of trials for both interventions and comparisons for IFX selection, along with the ranking heatmaps.


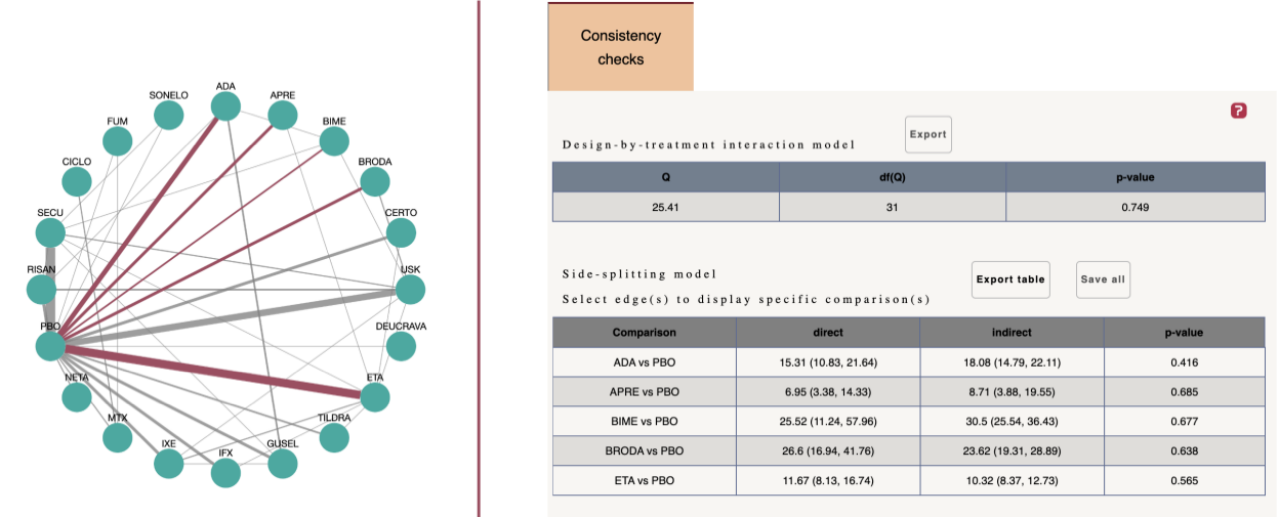


# **Figure S10.** The consistency checks results table, filtered by the selected edges within the diagram for the PASI90 outcome.


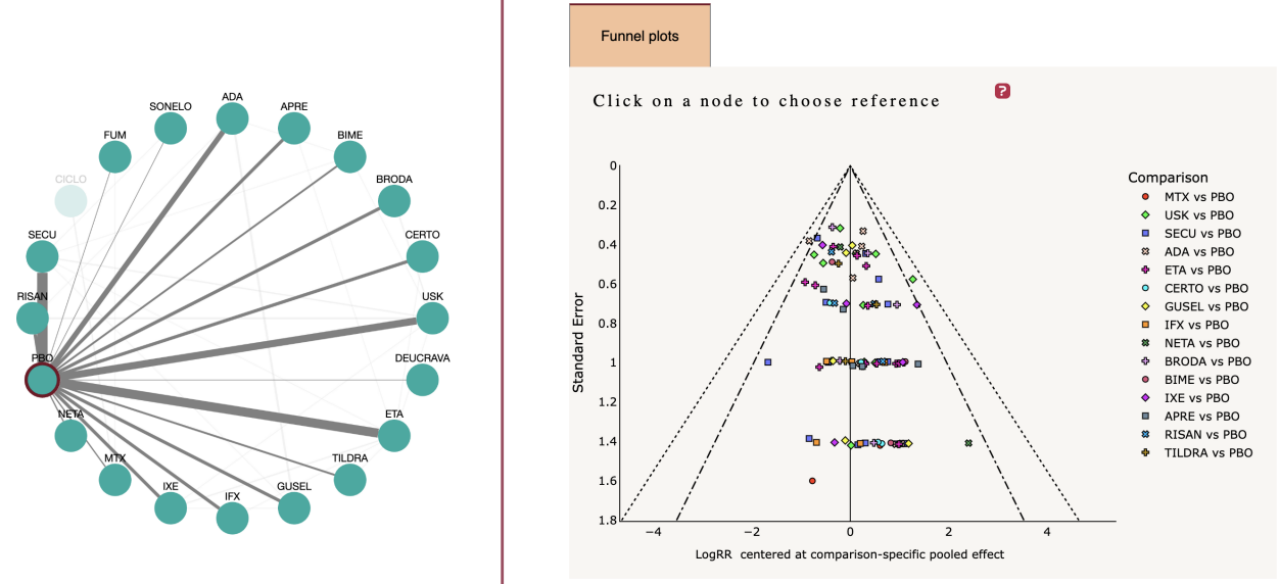


# **Figure S11.** The comparison-adjusted funnel plot for the PASI90 outcome. Clicking on the reference intervention within the diagram generates the funnel plot.


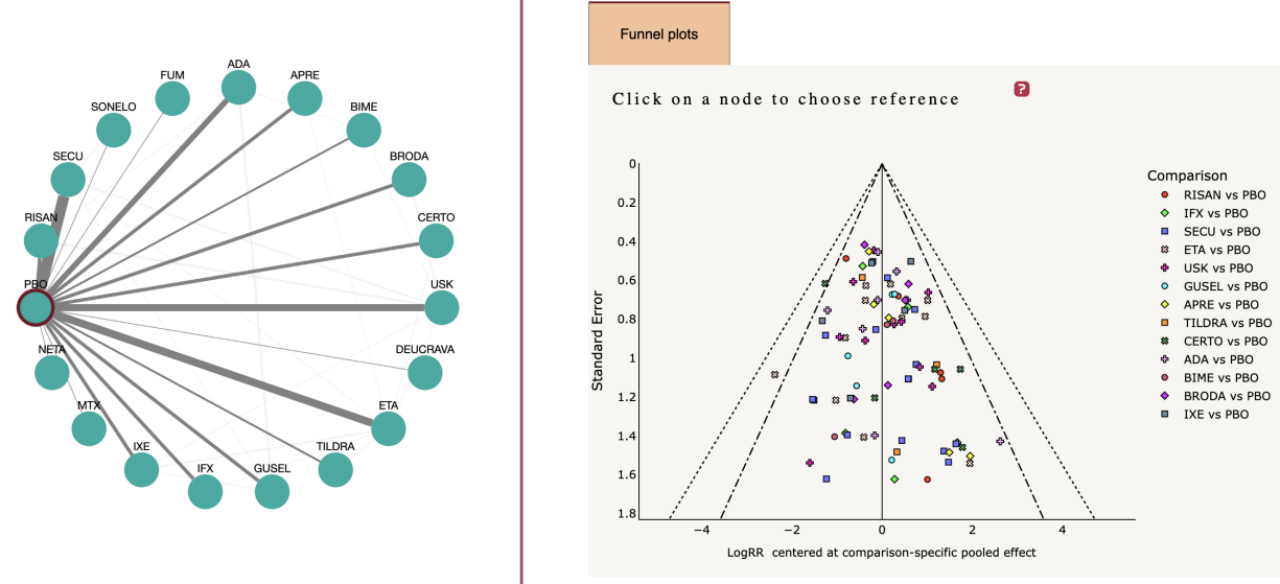


# **Figure S12.** The comparison-adjusted funnel plot for the SAE outcome. Clicking on the reference intervention within the diagram generates the funnel plot.


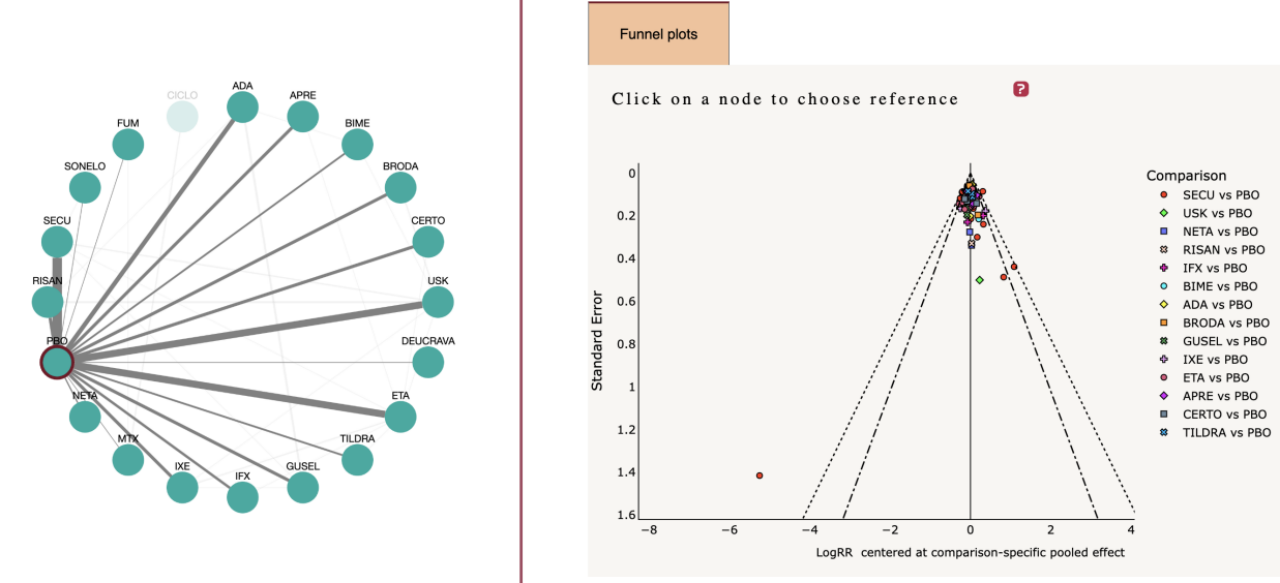


# **Figure S13.** The comparison-adjusted funnel plot for the AE outcome. Clicking on the reference intervention within the diagram generates the funnel plot.


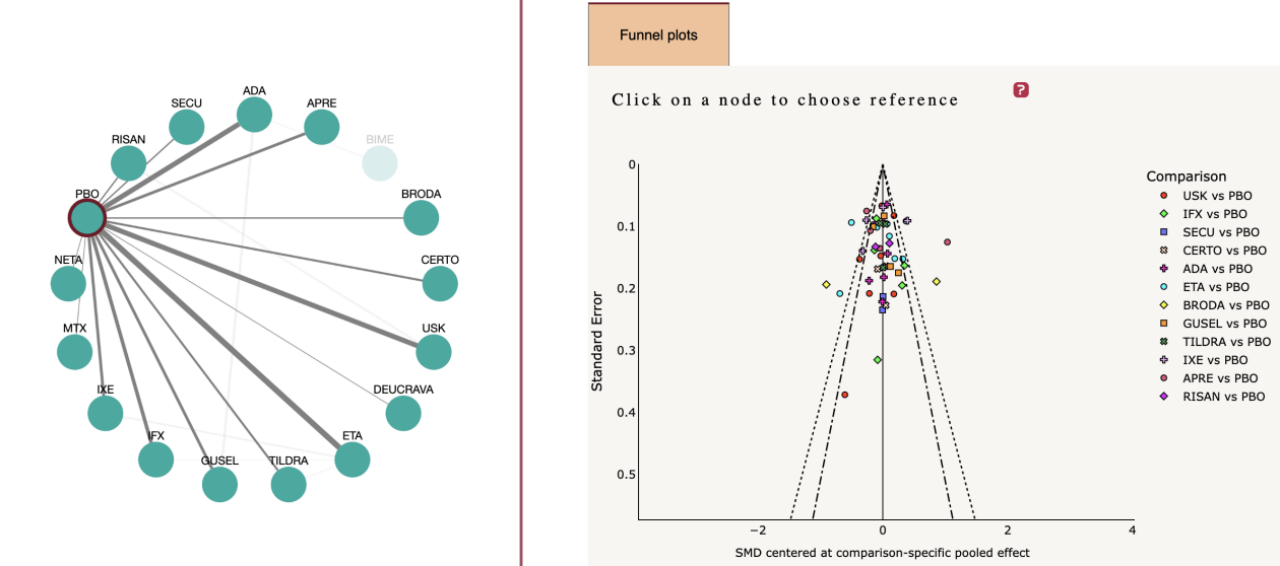


# **Figure S14.** The comparison-adjusted funnel plot for the DLQI outcome. Clicking on the reference intervention within the diagram generates the funnel plot.

# **
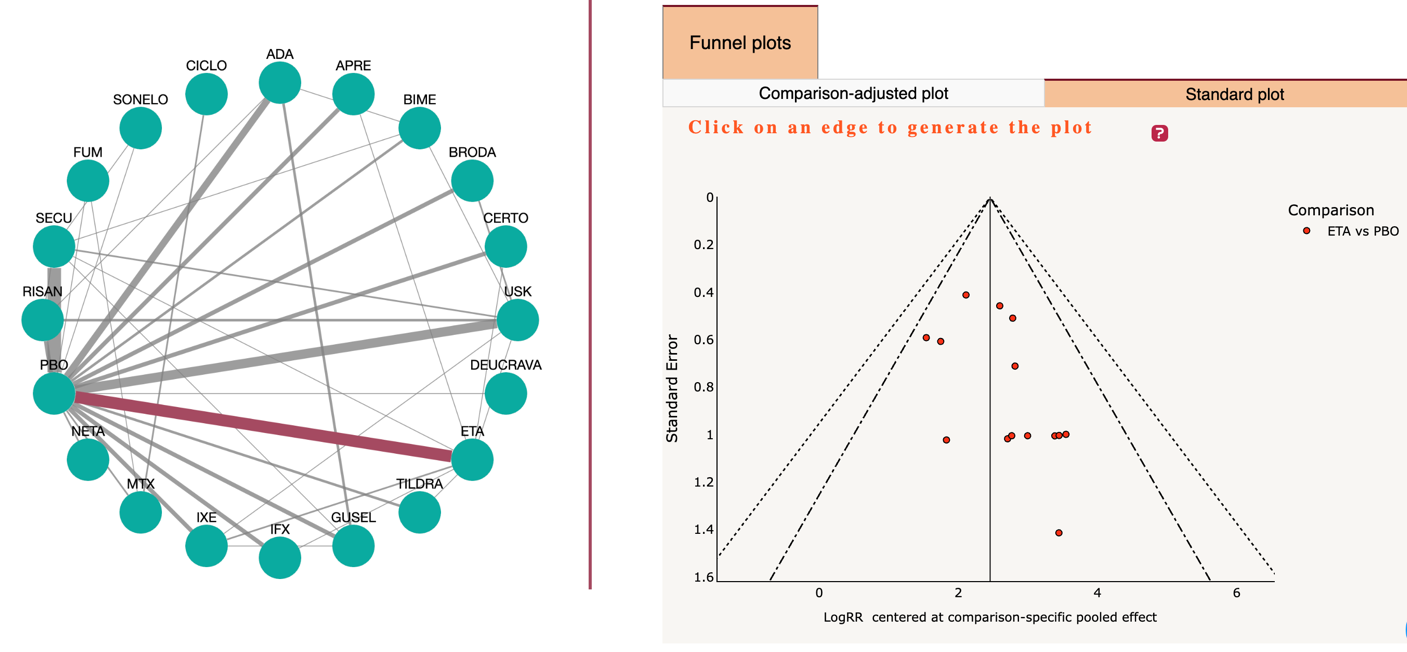
Figure S15.** The standard funnel plot of comparison ‘PBO VS ETA’ for the PASI90 outcome. Clicking on the edge within the diagram generates the funnel plot.


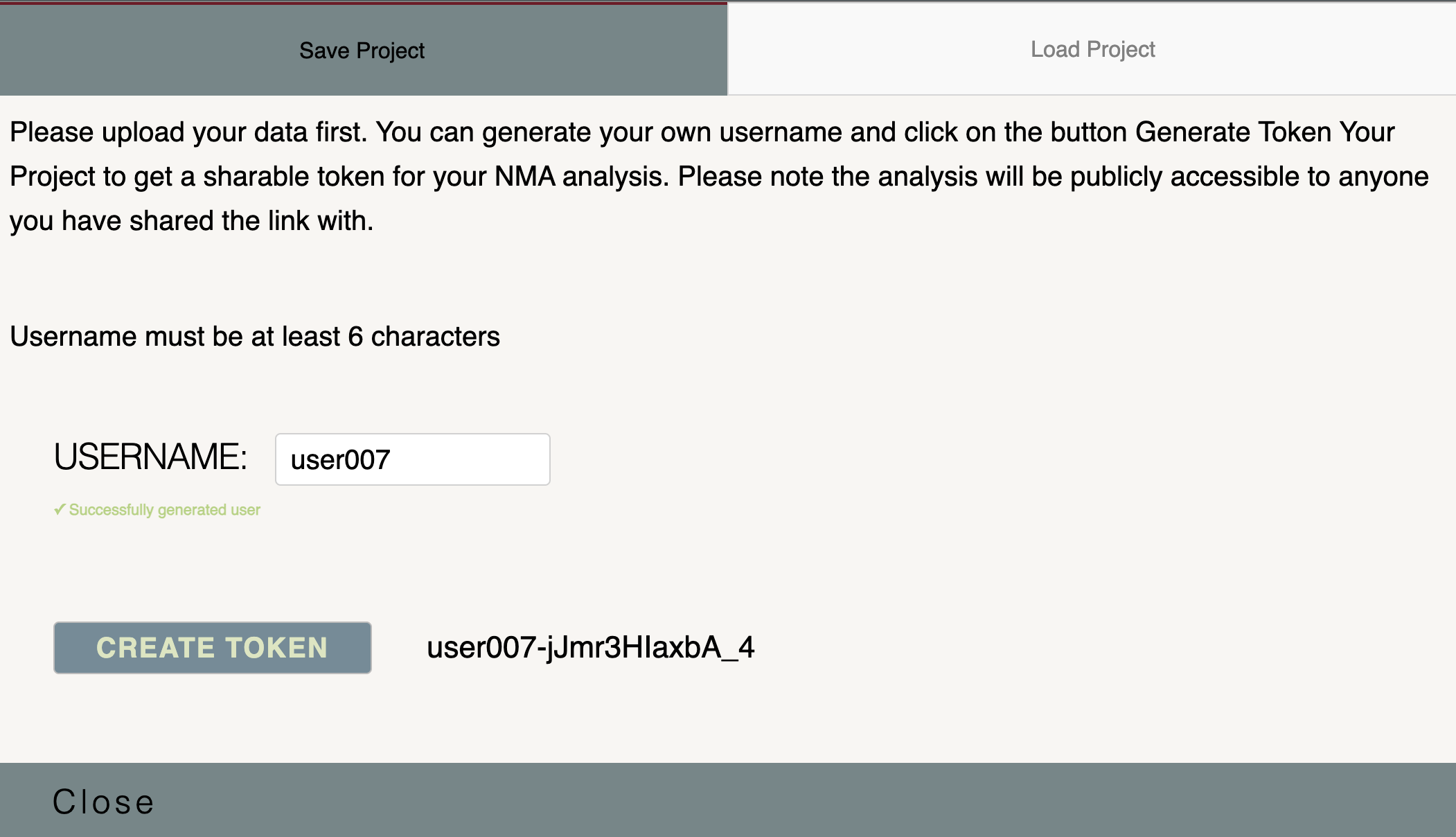


# **Figure S16.** Save the project by entering the username and click the CREATE TOKEN button.


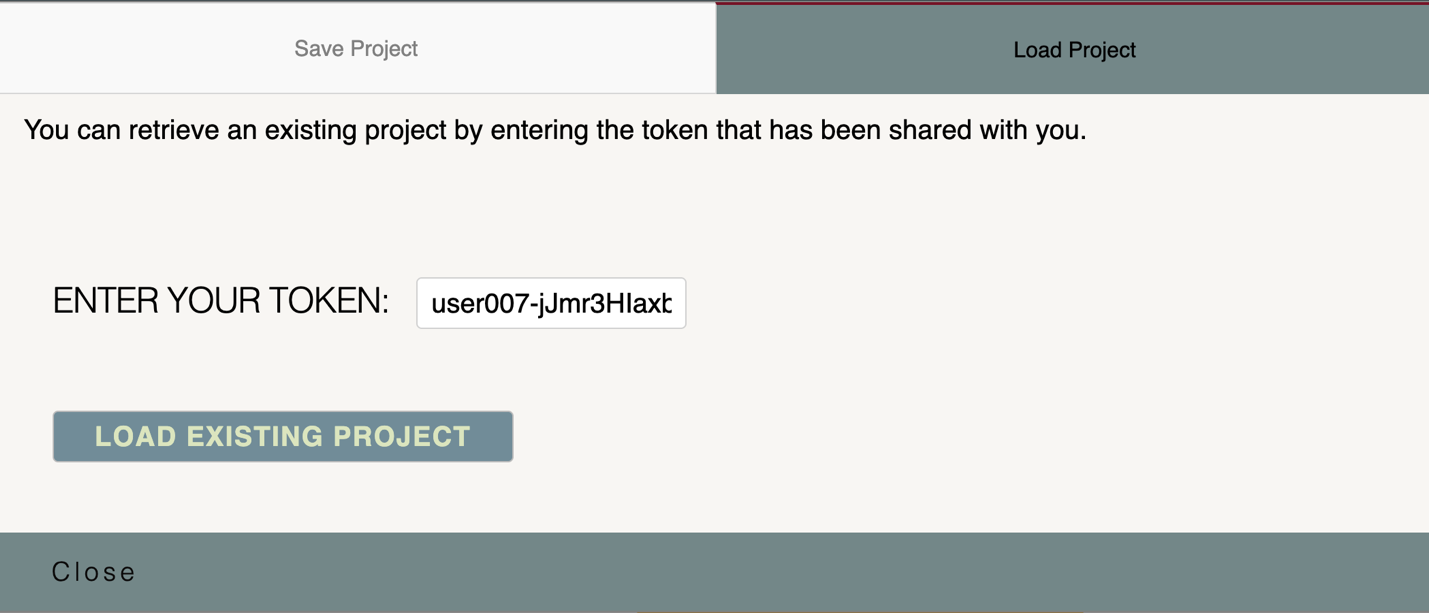


# **Figure S17.** Load the project by entering the token and click the LOAD EXISTING PROJECT button.
